# Dual-Targeting Nanoparticle-Mediated Gene Therapy Strategy for Hepatocellular Carcinoma by Delivering Small Interfering RNA

**DOI:** 10.3389/fbioe.2020.00512

**Published:** 2020-06-10

**Authors:** Qi Chang Zheng, Shuai Jiang, Yu Zhe Wu, Dan Shang, Yong Zhang, Shao Bo Hu, Xiang Cheng, Chen Zhang, Ping Sun, Yang Gao, Zi Fang Song, Min Li

**Affiliations:** ^1^Department of Hepatobiliary Surgery, Union Hospital, Tongji Medical College, Huazhong University of Science and Technology, Wuhan, China; ^2^Department of Vascular Surgery, Union Hospital, Tongji Medical College, Huazhong University of Science and Technology, Wuhan, China

**Keywords:** small interfering RNA, gene therapy, targeted therapy, chitosan, hepatocellular carcinoma, drug delivery

## Abstract

As a gene therapy strategy, RNA interference (RNAi) offers tremendous tumor therapy potential. However, its therapeutic efficacy is restricted by its inferior ability for targeted delivery and cellular uptake of small interfering RNA (siRNA). This study sought to develop a dual-ligand nanoparticle (NP) system loaded with siRNA to promote targeted delivery and therapeutic efficacy. We synthesized a dual receptor-targeted chitosan nanosystem (GCGA), whose target function was controlled by the ligands of galactose of lactobionic acid (LA) and glycyrrhetinic acid (GA). By loading siPAK1, an siRNA targeting P21-activated kinase 1 (PAK1), a molecular-targeted therapeutic dual-ligand NP (GCGA–siPAK1) was established. We investigated the synergistic effect of these two targeting units in hepatocellular carcinoma (HCC). In particular, GCGA–siPAK1 enhanced the NP targeting ability and promoted siPAK1 cell uptake. Subsequently, dramatic decreases in cell proliferation, invasion, and migration, with an apparent increase in cell apoptosis, were observed in treated cells. Furthermore, this dual-ligand NP gene delivery system demonstrated significant anti-tumor effects in tumor-bearing mice. Finally, we illuminated the molecular mechanism, whereby GCGA–siPAK1 promotes endogenous cell apoptosis through the PAK1/MEK/ERK pathway. Thus, the dual-target property effectively promotes the HCC therapeutic effect and provides a promising gene therapy strategy for clinical applications.

**Graphical Abstract:**
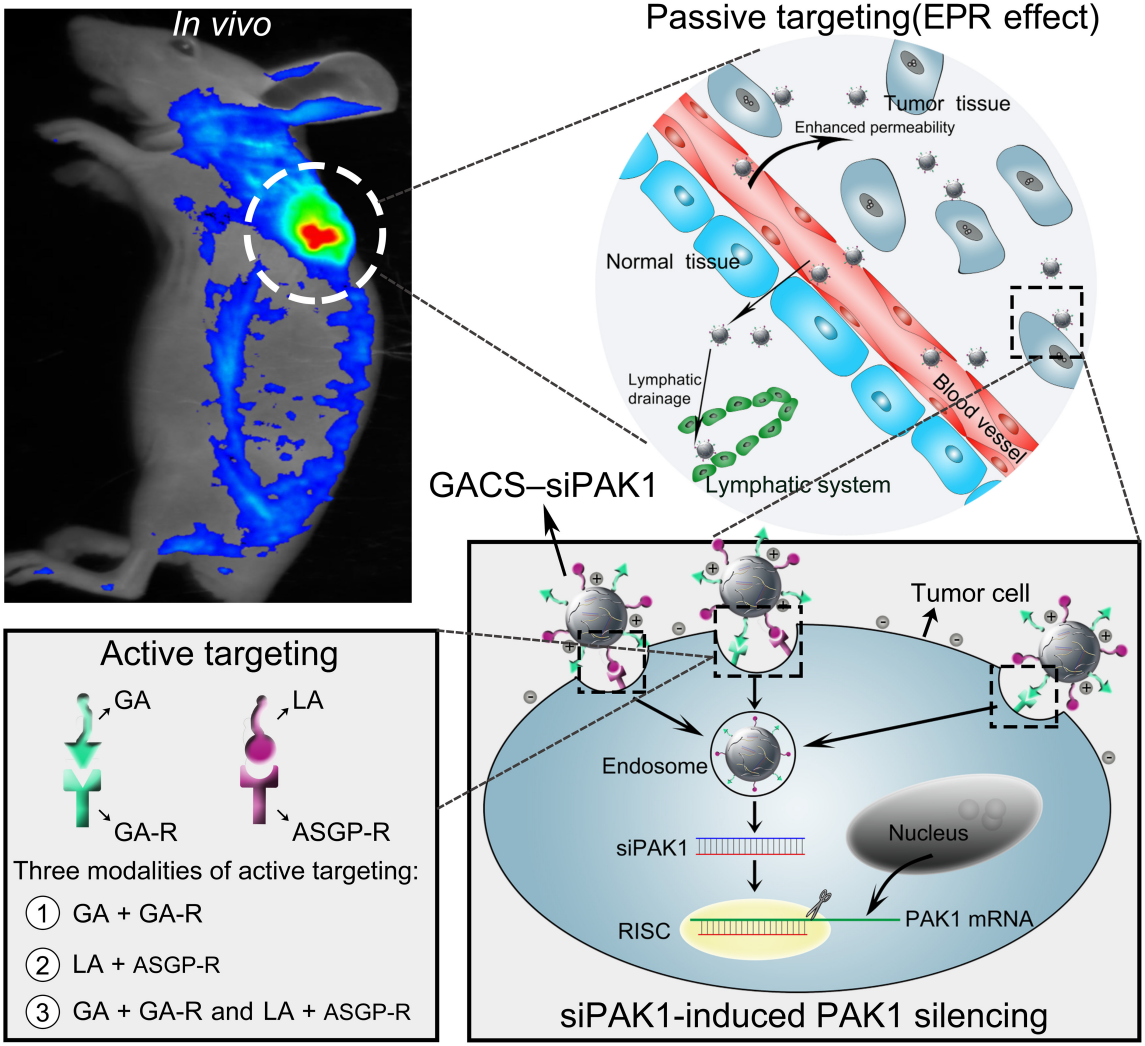


## Introduction

Hepatocellular carcinoma (HCC), as one of the most common cancers, is the fourth-leading cause of tumor-related deaths (Villanueva, 2019). Surgical therapies are the optimal option for patients with early-stage HCC. However, no ideal treatment exists for advanced-stage patients. Although Sorafenib (Llovet et al., 2008; Cheng et al., 2009; Zhu et al., 2015) and Lenvatinib (Kudo et al., 2018), as the only two chemotherapeutical drugs for first-line clinical treatment, apparently prolong the median survival of unresectable HCC patients, their application remains limited owing to various adverse events. Thus, a more precise, effective, and safe treatment is imperative.

As a precise gene therapy treatment strategy, RNA interference (RNAi) has attracted increasing attention in the recent years (Li et al., 2017; Jain et al., 2018; Uludag et al., 2019). RNAi-based therapeutics involve a double-stranded small interfering RNA (siRNA) composed of 21–22 nucleotides. The siRNA then motivates the sequence-specific enzymolysis effect of the target mRNA by means of complementary base pairing and suppresses the target gene expression (Tavernarakis et al., 2000; Elbashir et al., 2001). Consequently, this valuable approach circumvents the limitations of conventional systemic chemotherapy and offers a safe tumor therapeutic strategy, without toxic side effects (Chalbatani et al., 2019). However, as a type of oligonucleotide molecule, siRNA is restricted by its susceptibility to serum degradation and inferior cellular uptake ability when it is practically applied (de Wolf et al., 2007).

Chitosan (CS), which is a naturally occurring polysaccharide with effective biodegradability and biocompatibility, exhibits a strong ability to protect siRNA from serum degradation and deliver it to tumor cells (Ramesan and Sharma, 2012; Suarato et al., 2016). CS promotes the accumulation of anticancer macromolecules and drugs in solid tumors according to the enhanced permeability and retention (EPR) effect (a passive targeting process). Furthermore, an active targeting process, such as ligand-receptor-mediated endocytosis, can enhance cellular uptake of CS (Iyer et al., 2006; van der Meel et al., 2013; Li et al., 2016). However, the cellular uptake of single-ligand modified NPs will be restricted owing to the saturation phenomenon (attributed to the limited quantity of receptors on the target cell membrane). In this case, regardless of the extent to which the ligand ratio is elevated on the NP surfaces, the cellular uptake will not be increased (Kibria et al., 2011; Takara et al., 2012; Mei et al., 2014). To overcome this barrier and promote the cellular uptake efficacy, a dual-ligand targeted drug delivery system with the aim of reinforcing the tumor-targeting specificity has arisen (Jang et al., 2015; Mezghrani et al., 2015; Yang et al., 2015; Li et al., 2019).

In recent years, glycyrrhetinic acid (GA), a liver-targeting ligand, has been determined to recognize the glycyrrhetinic acid receptor (GA-R) overexpressed on hepatocytes, particularly hepatoma cells. Thus far, numerous studies have indicated that NPs decorated with GA may considerably increase the targeting capacity of HCC compared to those lacking it (Tian et al., 2010; Huang et al., 2011). The galactose of lactobionic acid (LA) is another effective ligand for the asialoglycoprotein receptor (ASGP-R), which is also overexpressed on the HCC cell membrane. Moreover, drug carriers containing LA have been developed to improve the targeting properties to hepatoma cells and the internalization of NPs (Huang et al., 2017; Pranatharthiharan et al., 2017). To further strengthen the targeting faculty for hepatoma cells, novel targeting-vehicle CS NPs decorated with dual-ligand GA and LA, namely GCGA NPs, were synthesized in our previous work, and demonstrated a significant tumor-targeting capability in HCC cells (Chen et al., 2012; Li et al., 2020). P21-activated kinase 1 (PAK1), one of the serine/threonine kinases, is widely overexpressed in various human cancers, including hepatic carcinoma. PAK1 affects various cellular phenomena, such as cell proliferation and migration, in normal and pathologic cells (Dummler et al., 2009). Thus, we speculate that the GCGA NPs entrapping siPAK1, which is a specific siRNA targeting and blocking PAK1 mRNA, may provide an effective treatment for HCC.

**Figure 1:**
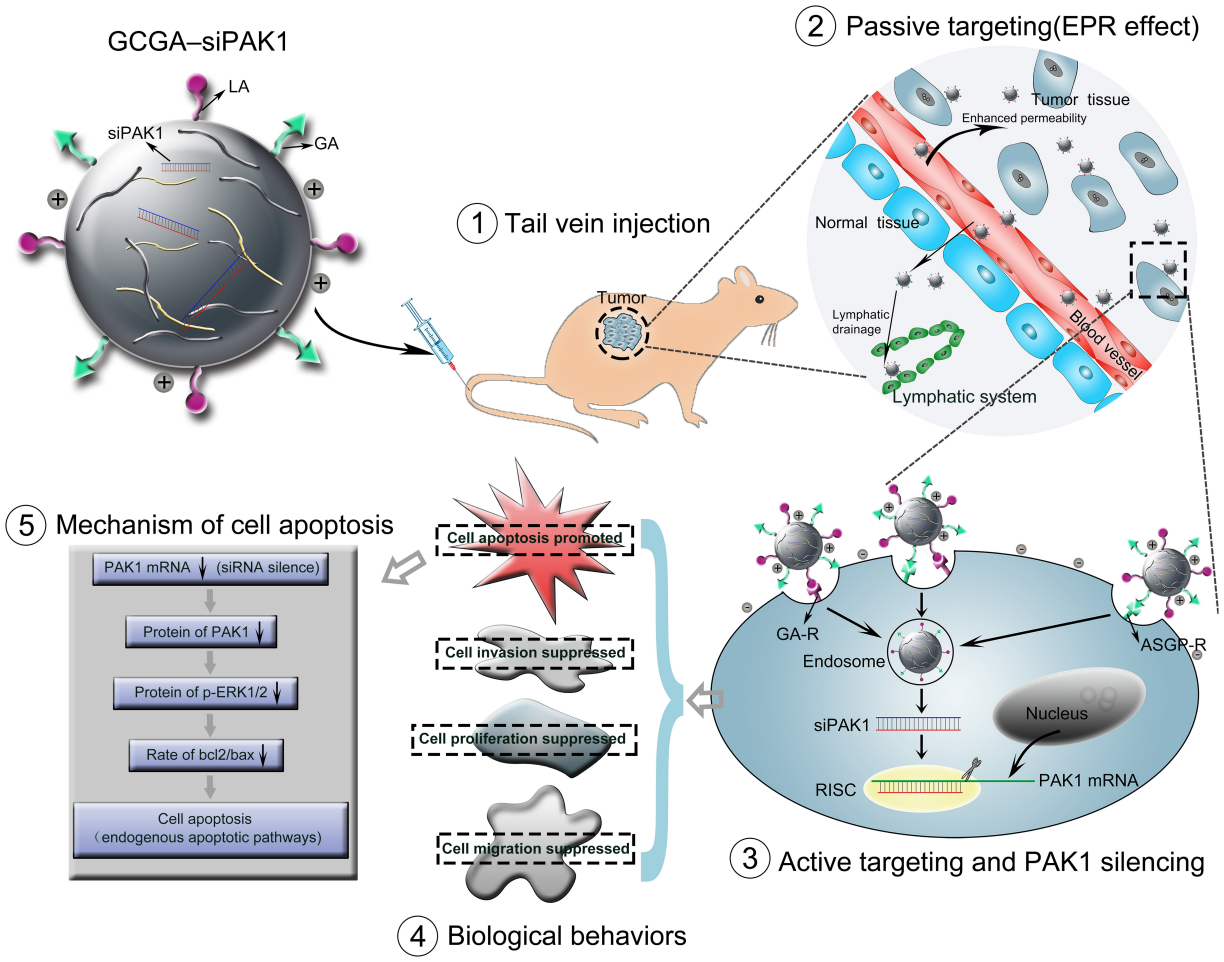
Schematic representation of GCGA–siPAK1 promoting targeted delivery and therapeutic efficacy in HCC xenograft mouse model. The process includes four steps: (1) intravenous administration of GCGA–siPAK1 *via* tail vein; (2) NPs accumulation in tumor tissue *via* passive targeting (commonly known as the EPR effect); (3) three modalities of active targeting *via* dual-ligand-receptor-mediated endocytosis and mechanism of RNAi (siPAK1-induced PAK1 silencing); (4) tumor biological behaviors after PAK1 silencing; and (5) molecular mechanism of promoting cell apoptosis *via* PAK1/MEK/ERK pathway.

In this study, we developed a novel dual-ligand targeted drug delivery system in which GCGA NPs entrapped siPAK1 (GCGA–siPAK1) for HCC therapy. Figure 1 illustrates the assumption that GCGA–siPAK1 enhances the siPAK1 cell uptake and influences tumor biological behaviors. Briefly, upon intravenous injection of GCGA-siPAK1 into a tumor-bearing mouse, these NPs can become selectively accumulated into the tumor interstitial fluid owing to the passive targeting (EPR effect). Then, the tumor cellular internalization of NPs will be facilitated by means of ligand-receptor-mediated endocytosis (the active targeting). As GA-R and ASGP-R receptors are both overexpressed on the surfaces of HCC cells, there are three modalities of active targeting: (1) the GA ligands bind with GA-R receptors; (2) the LA ligands bind with ASGP-R receptors; and (3) the GA and LA ligands bind with GA-R and ASGP-R receptors simultaneously. Subsequently, siPAK1 is released from the NPs and combines with the targeting PAK1 mRNA for PAK1 silencing. Thereafter, the expression of PAK1 is decreased, followed by the control of various tumor biological behaviors (including cell apoptosis, proliferation, invasion, and migration). Detailed experiments were performed to verify the feasibility of this assumption, investigate the molecular mechanism responsible for increased cell apoptosis, and assess the possibility of GCGA-siPAK1 for potential clinical applications.

## Materials and Methods

### Materials

The CS (deacetylation degree = 91%, viscosity = 78 mPas) was supplied by Aoxing Biotechnology Co., Ltd. (Zhejiang, China). The GA (purity > 98% by HPLC) was purchased from FUJIE Pharmaceutical Co., Ltd (Xi'an, China). The LA was obtained from Sigma-Aldrich (St. Louis, MO, USA). N-hydroxysuccinimide and 1-ethyl-3-(3-dimethylaminopropyl) carbodiimide hydrochloride were purchased from Shanghai Medpep Co., Ltd. (Shanghai, China). The other chemicals were of an analytical reagent grade. The siRNA of nonsense sequences (abbreviated as siNC), PAK1 siRNA (abbreviated as siPAK1; the sequence and potential binding site are presented in Supplementary Figure 1), FAM-labeled siRNA, was designed by Genepharma Co., Ltd. (Shanghai, China). Dulbecco's Modified Eagle's Medium (DMEM) and fetal bovine serum (FBS) were purchased from Gibco (Grand Island, NY, USA). A RIPA lysis buffer, WST-8 Cell Counting Kit, and one-step TUNEL fluorescence kit were obtained from Beyotime (Shanghai, China). A proteinase and phosphatase inhibitor, Tris-buffered saline/Tween 20 (TBST), 4% paraformaldehyde, and crystal violet were purchased from Servicebio (Wuhan, China). TRIzol reagent was purchased from Invitrogen (Carlsbad, USA). A PrimeScript RT Master Mix and Takara SYBR Green PCR Kit were purchased from Takara (Dalian, China). A BCA Protein Assay Kit was obtained from Boster Biological Technology, Ltd (California, USA). Polyvinylidene fluoride (PVDF) membranes were purchased from Millipore (Massachusetts, USA). The primary antibodies (including PAK1, p-ERK1/2, ERK1/2, bcl2, and bax) were all purchased from Cell Signaling Technology (Massachusetts, USA). The Horseradish peroxidase-conjugated secondary antibody was supplied by Proteintech (Wuhan, China). WesternBright ECL was purchased from Advansta (California, USA). An Annexin V-FITC/PI Apoptosis Detection Kit was purchased from Antgene Biotechnology (Wuhan, China). Transwell insert chambers with Matrigel were purchased from Corning (NY, USA). 12-O-tetradecanoyl phorbol-13-acetate (TPA) was obtained from Apexbio (Houston, USA). SCH772984 was purchased from Selleck Chemicals (Texas, USA).

### Synthesis and Characterization of GCGA

**Figure 2:**
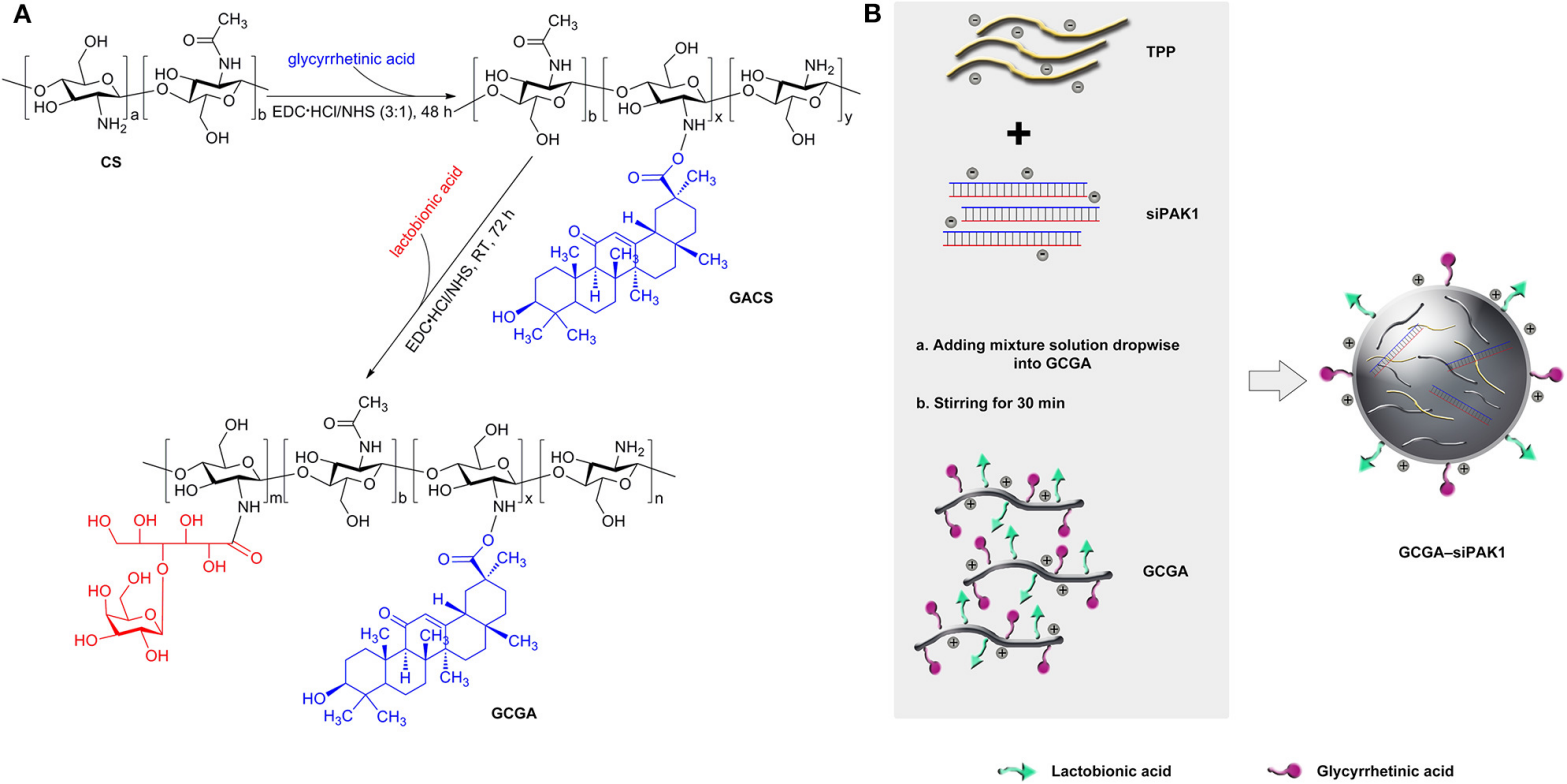
Schematic of dual-ligand GA and LA-modified CS NPs entrapped by siPAK1 (namely, GCGA–PAK1). **(A)** Synthetic route of GACS and GCGA. **(B)** Schematic of the fabrication of GCGA–siPAK1 by ionic gelation method.

As illustrated in Figure 2A, the GACS and GCGA were synthesized according to our previous study (Chen et al., 2012). In brief, the CS was modified with GA through crosslinks between the carboxyl groups of the GA and amino groups of the CS. Proton nuclear magnetic resonance (^1^H NMR) (D_2_O, 600 MHz) was conducted as follows: δ 4.26 (protons from the GA and CS moieties), 3.98 (protons from the GA and CS moieties), 3.28–3.12 (protons from the GA and CS moieties), 2.53 (protons from the CS moiety), 2.43–2.01 (protons from the CS moiety), and 1.43–1.42 (protons from the GA moiety). Thereafter, the GA-modified CS (GACS) was decorated with LA to prepare the GCGA product. ^1^H NMR (D_2_O, 600 MHz) was conducted as follows: δ 4.34 (protons from the LA and GA moieties), 4.00–3.99 (protons from the LA moiety), 3.66–2.97 (protons from the LA, GA, and CS moieties), 2.63 (protons from the CS moiety), and 2.11–2.02 (protons from the LA and CS moieties).

### Preparation and Characterization of GCGA–siNC, GCGA–siPAK1, GACS–siPAK1, and CS–siPAK1 NPs

The GCGA–siNC, GCGA–siPAK1, GACS–siPAK1, and CS–siPAK1 were produced by ionic gelation, with the compositions enumerated in Table 1. The CS polymers with a positive charge were dissolved in a sodium acetate buffer (3%, HAC) to obtain a 1 mg/mL working solution. Tripolyphosphate (TPP) was selected as an ionic crosslinker to connect the CS polymers (Abdelrahman et al., 2017). Then, 0.25 mL of siRNA (siPAK1 or siNC) in diethyl pyrocarbonate water (30 nmol/mL) was added to 1.0 mL of a TPP aqueous solution (0.5 mg/mL) for the mixture solution while stirring for 1 h. As illustrated in Figure 2B, the GCGA–siPAK1 was fabricated by dropwise adding of the mixture solution of the siRNA and TPP to 1.75 mL of the GCGA, GACS, and CS solutions (1 mg/mL, at a CS-to-TPP weight ratio of 4.7:1) under constant magnetic stirring (1,300 rpm) for 30 min at 25°C. The final NP concentration was ~0.75 mg/mL. The final siRNA concentration was ~2.5 × 10^3^ nM.

**Table 1:** Compositions of various NPs.

**NPs**	**Delivery vehicle**	**Entrapped nucleotides**
GCGA–siNC	Dual-ligand LA and GA-modified CS	siNC
GCGA–siPAK1	Dual-ligand LA and GA-modified CS	siPAK1
GACS–siPAK1	Single-ligand GA-modified CS	siPAK1
CS–siPAK1	CS without ligand	siPAK1

The sizes of the GCGA–siNC, GCGA–siPAK1, GACS–siPAK1, and CS–siPAK1 were measured using dynamic light scattering (DLS). The zeta potential was determined by a Zetasizer 3000HS (Malvern Instruments, Worcestershire, UK). The morphology was obtained by using a transmission electron microscope (TEM; Hitachi H-7000FA, Tokyo, Japan). The *in vitro* release experiments were administrated at 37°C in phosphate buffer saline (PBS, 0.01 M) with pH = 5.0 and 7.4 for a period of 96 h, respectively. The siRNA-loaded NPs (at an NP concentration of 0.3 mg/mL) were incubated in a rotary shaker (100 rpm) at 37°C. For each time point, the samples were removed and treated as described by Jensen et al. (2012). The extracted siRNAs were determined by a NanoDrop1000 Spectrophotometer (Thermo Fisher Scientific, Massachusetts, USA). The encapsulation efficiency (EE) values were measured by the same method and calculated according to the equation: EE% = [actual siRNA loading/theoretical siRNA] × 100%.

### Cell Culture

The human HCC cell lines (Hep3B and HepG2) were purchased from the Cell Resource Center, Shanghai Institute of Cell Biology, Chinese Academy of Sciences, and cultured with DMEM supplemented with 10% FBS and 100 U/mL Penicillin-Streptomycin at 37°C in a humidified incubator containing 5% CO_2_.

### *In vitro* Cellular Uptake of NPs

The cells were seeded in 24-well plates and cultured for 12 h. Then, 25 μL of the NPs were added into a 475 μL culture medium (final siRNA concentration of ~120 nM). After incubating for 4 h, the cells were washed using cold PBS and fixed with 4% paraformaldehyde for 20 min. Finally, the sample was observed under a fluorescent microscope (Olympus, Tokyo, Japan). The influence intensity was measured by ImageJ software for statistical analysis.

### Hemolysis Assay

Fresh blood (2 mL) was prepared by cardiac puncture from BALB/c mice and diluted with 4 mL PBS. The red blood cells (RBCs) were then separated from the serum by centrifugation at 3 × 10^3^ rpm for 10 min. After careful rinsing three times with 5 mL of saline solution, a suspension of RBCs was added into the saline solution (negative control), deionized water (positive control), and various NP solutions at a double concentration of treatment. After being mixed completely, all samples were incubated at 37°C for 1, 2, and 3 h. At each time point, every sample was re-suspended and 10 μL was sucked out onto a glass slide to observe the morphological changes in the erythrocyte by means of a phase contrast microscope (Olympus, Tokyo, Japan). Thereafter, the mixtures were centrifuged at 1.2 × 10^4^ rpm for 10 min and the hemolysis images were captured. Finally, the supernatants (100 μL) of each tube were transferred to a 96-well plate, and the optical density at 570 nm was measured by a microplate reader (Thermo Fisher Scientific, USA).

### Animal Model

All mouse experiments complied with the guidelines of the Animal Care Committee at Tongji Medical College. Female BALB/c nude mice weighing 17–19 g were obtained from the Vital River Laboratory Animal Technology Co. (Beijing, China) and housed in a pathogen-free animal facility. To establish the Hep3B tumor model, all nude mice were inoculated subcutaneously with 2 × 10^6^ cells/mouse in the right backside. The tumor volumes were evaluated by a caliper, and calculated as follows: volume = (tumor width)^2^ × (tumor length)/2.

### *In vivo* Imaging of NPs in Hep3B-Xenografted Nude Mice

The BALB/c nude mice bearing tumors were established as described above. The mice with a similar tumor size were randomly divided into five groups (*n* = 3) and intravenously injected with saline, GCGA–siNC, GCGA–siPAK1, GACS–siPAK1, and CS–siPAK1 through the tail vein (100 μL of each NP and a final siRNA concentration of ~120 nM in the peripheral circulation). The saline group was set as the control. After administration for 8 h, the mice were anesthetized using 100 μL of 10% chloral hydrate and *in vivo* real-time fluorescence imaging was conducted by an *in-vivo* FX PRO (Bruker, Germany) (λex = 490 nm and λem = 535 nm). Thereafter, the mice were sacrificed, and the major organs were harvested. The fluorescence intensity of the tumor tissues and various organs of each group (*n* = 3 in each group) was measured.

### Cell Viability Assay

The cell viability was demonstrated using a WST-8 cell counting kit, according to the manufacturer's protocol. Briefly, the cells were seeded in 96-well plates at a density of 2 × 10^3^ cells/well. Following attachment, appropriate treatment with the NPs was performed (final siRNA concentration of ~120 nM) for 48 h. Then, 100 μL of the fresh culture medium, including a 10 μL CCK8 solution, was added into each well. The cells were subsequently incubated for an additional 1 h at 37°C in the dark. Finally, the optical density (OD) at 450 nm was measured using a microplate reader (Thermo Fisher Scientific, Massachusetts, USA).

### Cell Density and Colony-Forming Assay

The cells were seeded at a density of 1.2 × 10^5^ cells/well onto six-well plates. Following attachment, the cells in each group were treated with the NPs (final siRNA concentration of ~120 nM) for 48 h. Then, the cells were digested using trypsin and 2 × 10^3^ cells were seeded onto additional six-well plates. After 7 days, the cells were fixed with 4% paraformaldehyde and stained with 0.4% crystal violet. Thereafter, the numbers of colonies of more than 50 cells were observed and recorded under a microscope. The colony-formation ability was assessed by means of the colony-forming efficiency (CFE): CFE = [numbers of colonies/numbers of seeded cells] × 100%.

### Cell Apoptosis

The cell apoptosis was determined using flow cytometry and TUNEL assays. Firstly, the cells were inoculated in 12-well plates and allowed to attach overnight, followed by treatment with the NPs (final siRNA concentration of ~120 nM) for 48 h. Thereafter, for the flow cytometry assays, according to the instructions of the Annexin V-FITC/PI Apoptosis Detection Kit, the cells were digested with ethylenediaminetetraacetic acid-free trypsin and pelleted by centrifugation at 1,500 rpm for 5 min at 25°C. The cells were washed twice using cold PBS and re-suspended in 195 μL of 1 × binding buffer at a concentration of 1 × 10^6^ cells/mL. Then, 5 μL Annexin V-FITC was added to each tube and these were incubated at 25°C for 15 min in the dark. Subsequently, 10 μL of PI was added and incubation took place for 5 min before the samples were analyzed with FACSCalibur flow cytometry (Becton Dickinson, New Jersey, USA). For the TUNEL assays, the cells were washed with cold PBS and fixed with 4% paraformaldehyde for 30 min. Subsequently, the cells were permeabilized using 0.1% Triton X-100, followed by treatment with the TUNEL cell apoptosis detection kit for 1 h at 37°C. The FITC-labeled positive cells were captured under a fluorescent microscope (488 nm excitation and 530 nm emission; Olympus, Tokyo, Japan).

### Cell Invasion Assay

The cell invasion was analyzed using 24-well Transwell insert chambers with Matrigel. After treatment with the NPs (final siRNA concentration of ~120 nM) for 48 h, the cells with 8 × 10^4^ were re-suspended in a 300 μL serum-free medium and seeded in the upper chamber. The lower chambers were filled with 700 μL medium with 10% FBS. Following incubation for 24 h, the non-invasive cells remaining on the upper surface of the chamber were removed using a cotton swab. The invasive cells on the lower surface were fixed with 4% formaldehyde for 30 min and stained with 0.5% crystal violet for 30 min. Finally, the labeled invasive cells were photographed under a microscope at × 200 magnification.

### Wound Healing Assay

Following treatment with the NPs (final siRNA concentration of ~120 nM) for 48 h, the cells were digested and seeded into 12-well plates at 1 × 10^6^ cells/well and incubated overnight. After the cells adhered and spread completely, a p200 pipet tip was used to create a scratch. Thereafter, the plates were washed three times with PBS and cultured using the serum-free medium. The cells were photographed at each time point, and the scratch area was measured by ImageJ software (National Institutes of Health, Bethesda, MD, USA).

### Quantitative Real-Time PCR (RT–qPCR)

The total RNA of each group was extracted using a Trizol reagent and quantified by a NanoDrop ND-1000 spectrophotometer. The first strand cDNA was synthesized by reverse transcription with PrimeScript RT Master Mix. Thereafter, a Takara SYBR Green PCR Kit was used and RT–qPCR was performed on a StepOnePlus Real-Time PCR system (Applied Biosystems, California, USA). The relative mRNA abundance of the PAK1 gene was normalized using glyceraldehyde-3-phosphate dehydrogenase (GAPDH) and analyzed by the 2^−ΔΔCT^ method (Livak and Schmittgen, 2001). The primer sequences of the PAK1 are listed as follows: Forward, 5′-GCTACAGGTGAGAAAACTGAGGT-3′; Reverse, 5′-TTCAATGCTGGACACACGGT-3′.

### Western Blot

For total protein isolation, the tumor tissues and cell samples were both lysed in a RIPA lysis buffer containing a proteinase and phosphatase inhibitor. Then, the lysates were centrifuged at 12,000 rpm at 4°C for 10 min. The protein concentrations were determined by a BCA Protein Assay Kit. Equivalent amounts of pre-denatured protein from each sample were separated in 10% SDS–PAGE polyacrylamide gels and electrotransferred to PVDF membranes. After incubation with TBST containing 5% skim milk for 1 h at 25°C, the membranes were incubated with the appropriate primary antibody overnight at 4°C. After three sets of 10 min of washing using TBST, the membranes were incubated with the horseradish peroxidase-conjugated secondary antibody for a further 1 h at 25°C. After three consecutive washings, the protein bands were visualized by the Western Bright ECL using a ChemiDoc XRS+ photo-image system (Bio-Rad, California, USA). The signal intensity was quantified using ImageLab (Bio-Rad, California, USA).

### *In vivo* NP Efficacy

The BALB/c nude mice bearing Hep3B cells were selected for examining the effect of the NPs *in vivo*. When the tumors bearing on the BALB/c nude mice reached a volume of ~100 mm^3^, the mice were randomly divided into four groups. Each group was injected with 100 μL GCGA–siNC, GCGA–siPAK1, GACS–siPAK1, and CS–siPAK1 solution through the tail vein. On approximately the third day after different treatments, mice from each group (*n* = 4) were sacrificed and the tumor tissues were excised. These tissues were embedded in paraffin and sliced. To confirm the apoptosis efficacy of the NPs *in vivo*, slices of the tumor tissues were stained with hematoxylin–eosin (H&E) and assessed using the TUNEL assay. Cell extracts of xenografted tumor tissues from all groups (*n* = 4 in each group) were prepared in RIPA lysates with a proteinase and phosphatase inhibitor. The protein concentration was measured by the BCA Protein Assay Kit and the protein expression was evaluated by the western blot, as described previously. To record the survival time, the remaining mice (*n* = 10 in each group) were treated with the same NPs every week. Moreover, the tumor volume was measured every 2 days for 16 days.

### TUNEL Assay for Tumor Slices

The apoptosis levels of the tumor tissues after treatment were determined using a one-step TUNEL fluorescence kit. The experiment was performed according to the manufacturer's recommended procedure. In brief, sections of the tumor tissues were incubated with the TUNEL reaction mixture for 1 h at 37°C. Thereafter, the sections were stained with DAPI for 10 min to localize the cell nuclei. The apoptotic cells were imaged under a fluorescence microscope (Olympus, Tokyo, Japan).

### *In vivo* Biosafety Studies

To assess the toxicities of the GCGA–siPAK1 *in vivo*, healthy BALB/c nude mice were assigned into four groups (*n* = 4 in each group). The mice were intravenously injected with 200 μL of saline (used as a control), GCGA–siNC, GCGA–siPAK1, GACS–siPAK1, and CS–siPAK1 (a double dose for treatment) every week. The clinical conditions of all the mice were observed and the weights were measured daily. On the day 8, the mice were sacrificed and their blood samples were taken, through cardiac puncture, for blood chemistry tests. Thereafter, the main organs of the mice were excised and stained using H&E for histological observations.

### Statistical Analysis

All data were expressed as the mean ± standard deviation (SD). For analysis between two groups, the results were evaluated by the Student's *t*-test. For comparisons among multiple groups, results were obtained by one-way analysis of variance with Student–Newman–Keuls as a *post-hoc* test. To compare the Kaplan–Meier survival curves, the results were analyzed by the log-rank test. SPSS version 10.0 was used for all analyses, and *P* < 0.05 was considered as statistically significant. All experiments were performed at least three times.

## Results and Discussion

### Characterizations of NPs

**Figure 3:**
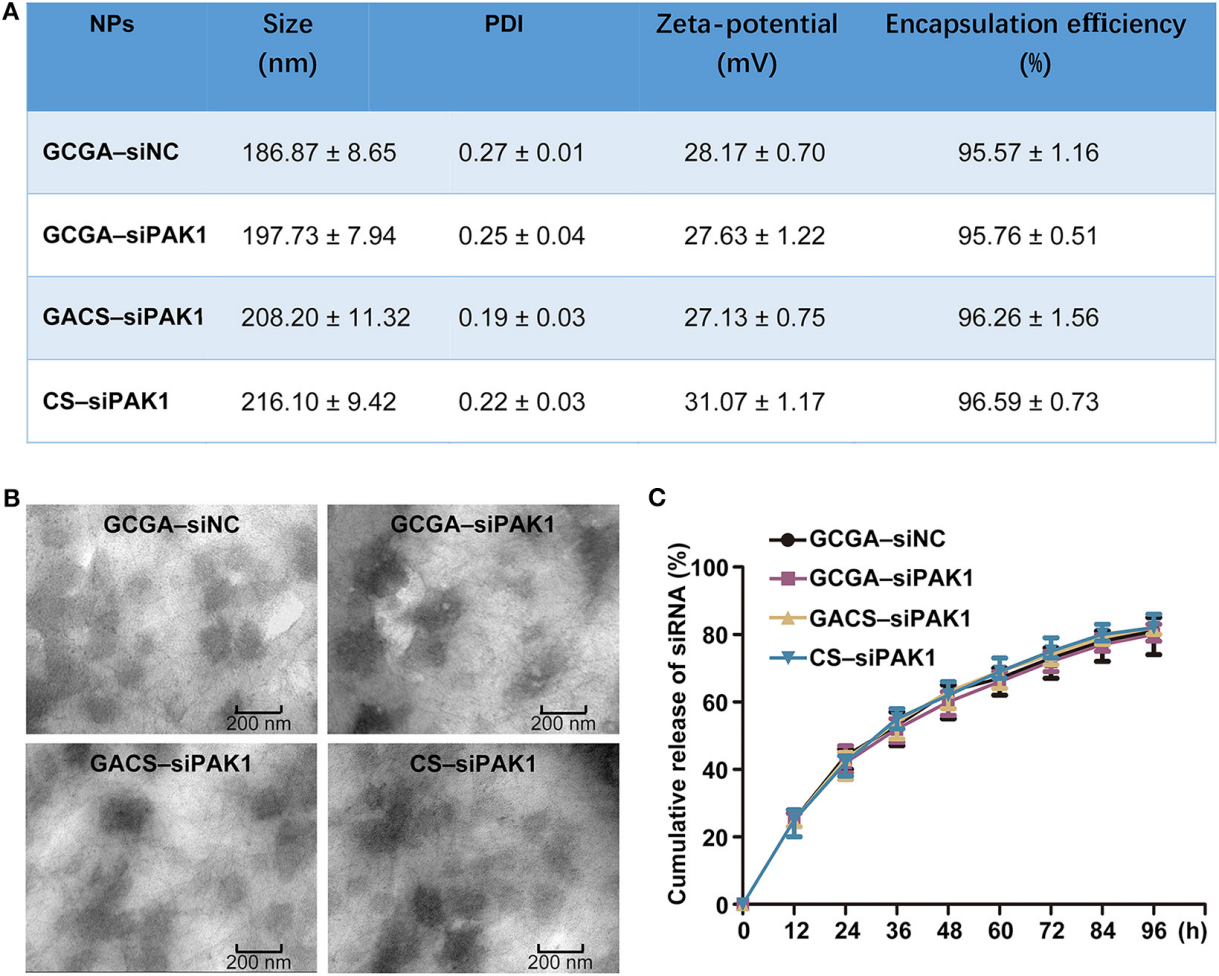
Characterizations of NPs. **(A)** Size, zeta potentials and encapsulation efficiencies of NPs. Data represents the mean ± SD (*n* = 3). PDI, polydispersity index. **(B)** Representative TEM images of NPs. **(C)** Release profiles of siRNA from all NPs in pH = 5.0 and 7.4, respectively. Data represents the mean ± SD (*n* = 3).

As illustrated in Figure 3A, the size distribution was measured by DLS. The average diameters of the GCGA–siNC, GCGA–siPAK1, GACS–siPAK1, and CS–siPAK1 were 186.87, 197.73, 208.20, and 216.10 nm, respectively, with a narrow size distribution. These average sizes were intermediate between 175 and 380 nm, making it difficult for them to pass through the hepatic sinusoid, yet easily pass through extensive angiogenesis present in a hepatic carcinoma (Ballet, 1990; Hobbs et al., 1998). Thus, we demonstrated that these NPs are theoretically ideal for HCC treatment. Moreover, the TEM images revealed that all NPs were thoroughly dispersed with a regular spherical shape (Figure 3B). Spherical NPs are generally considered to be superior to those that are rod-shaped, owing to the cell membrane taking longer to wrap elongated formulations (Verma and Stellacci, 2010). Further, the average size of the NPs captured by the TEM was ~150 nm. These were smaller than those measured by DLS owing to the shrinkage of the NPs *via* drying under high vacuum. The average zeta potentials of all NPs were positive (approximately greater than +27 mV) owing to the abundant amino groups on the CS backbones. This positive charge character may serve to promote the interaction between the NPs and tumor cell membrane, which are often negatively charged, and eventually increase the permeability and bioavailability of the NPs (Verma and Stellacci, 2010; Li et al., 2013; Yuan et al., 2014). Additionally, the encapsulation efficiency of the NPs was ~96%. Figure 3C depicts the cumulative release profiles of siRNA in 0.1 M PBS at pH = 5.0 and 7.4. Obtaining similar results in all groups indicates that the additional modifications in the CS (including the single-ligand, GA, and dual-ligands, GA and LA) does not likely influence the accumulated release of the siRNA.

### Enhanced Cellular Uptake of GCGA–siPAK1 *in vitro* and *in vivo*, and Hemocompatibility

**Figure 4:**
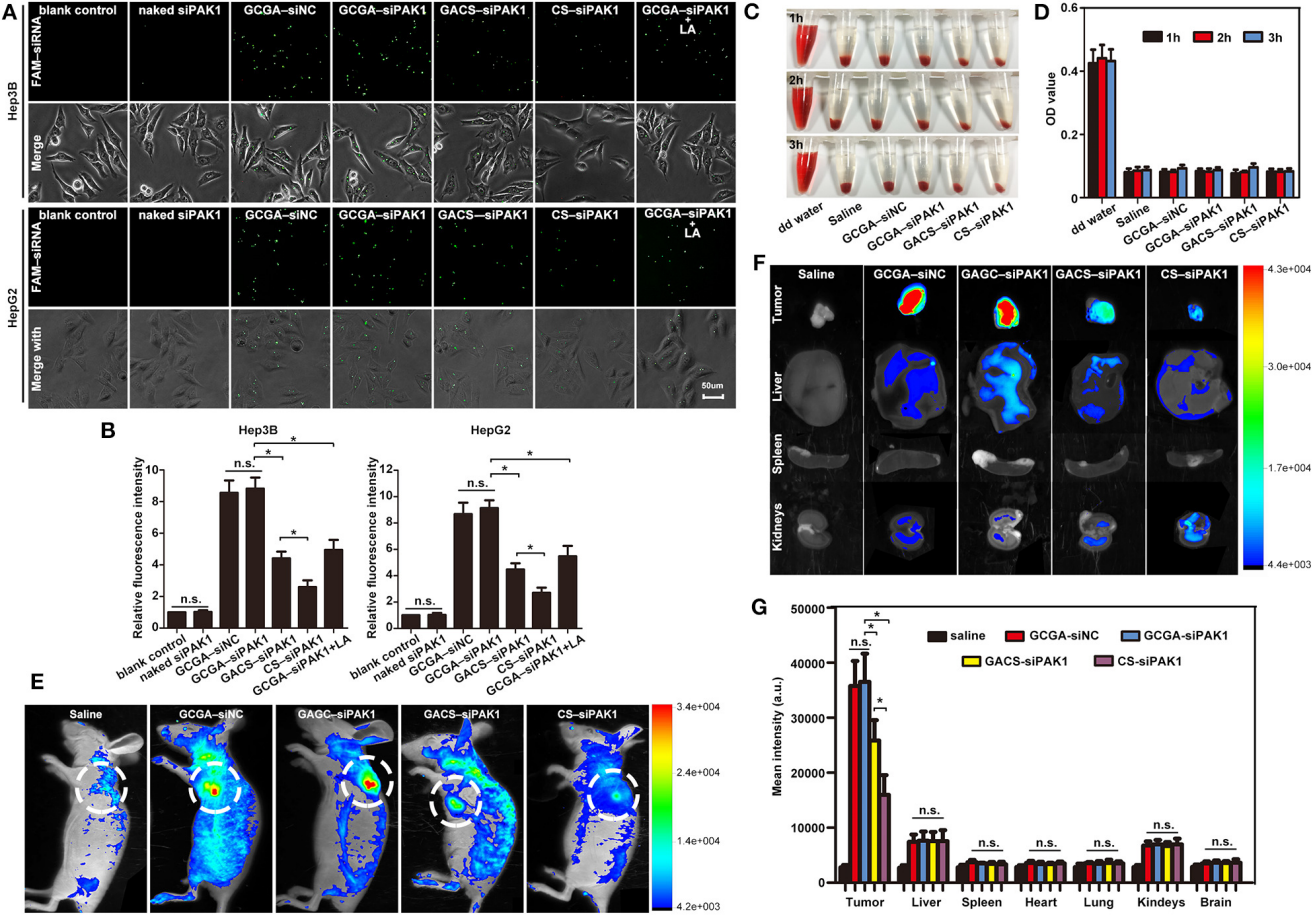
Enhanced cellular uptake of GCGA–siPAK1 *in vitro* and *in vivo*, and hemocompatibility. **(A)** Fluorescence images of HCC cells incubated with GCGA–siNC, GCGA–siPAK1, GACS–siPAK1, and CS-siPAK1 at a final FAM-labeled siRNA concentration of 120 nM for 4 h under the same conditions; and fluorescence images of GCGA–siPAK1 in HCC cells pretreated with free LA (100 μg/mL) for 30 min (in the seventh column). **(B)** Statistical analysis of fluorescence intensity according to **(A)**. **(C)** Photographs displaying mixtures of RBCs with NPs after sample centrifugation. **(D)** Quantitative results of hemoglobin in supernatant. **(E)** Biodistribution of NPs in tumor-bearing mice treated with siRNA-loaded NPs (equivalent siRNA; final siRNA concentration of 120 nM) through tail vein injection. Images were captured at 8 h following injection. White circles indicate tumor sites. **(F)** *Ex vivo* fluorescence images of tumor tissue and various organs from mice injected with NPs. The mice were sacrificed 8 h after injection. **(G)** Statistical analysis of fluorescence intensity according to **(F)**. The data represent the mean ± SD (*n* = 3). ^*^P < 0.05; NS, not significant.

Fluorescent microscopy was used to investigate the cellular uptake of the NPs *in vitro*. Compared to the blank control group, the cells treated with naked siPAK1 did not exhibit a fluorescence signal, while the other groups all displayed fluorescence signals with different intensities (Figures 4A,B). These results verify that the naked siPAK1 is degraded by nucleases and does not efficiently enter the cytoplasm (Ramesan and Sharma, 2012). Furthermore, all NPs in this study elicit beneficial effects in the protection and transportation of siRNA. However, only a weak fluorescence signal was observed in the cells incubated with CS–siPAK1, while the fluorescence signal was significantly elevated in those with GACS–siPAK1. These results indicate that the NPs decorated with the single-ligand GA exhibit a superior targeting capacity to those lacking the ligand. More interestingly, a higher fluorescence signal was obtained in the cells with GCGA–siPAK1 relative to GACS–siPAK1, indicating that the dual-ligand NP, GCGA–siPAK1, apparently enhances the targeting capability and carries more target siRNA into the HCC cells. The cells with GCGA–siNC exhibited a similar fluorescence intensity to GCGA–siPAK1, demonstrating that the GCGA–siNC and GCGA–siPAK1 both exhibit the greatest efficacy in terms of the targeting and transportation of siRNA. Moreover, different siRNA nucleotide sequences did not disturb the targeting and carrying ability of the NPs. Compared to the single-ligand NPs, those containing dual-ligands were co-modified with an additional ligand, LA, which was the only unique chemical component in the structure. Hence, we postulated that the targeting superiority of the dual-ligand NPs was likely caused by the specific LA–ASGP-R-mediated endocytosis in the HCC. To verify this hypothesis further, an LA competitive inhibition assay (in which free LA competes with GCGA–siPAK1 for ASGP-R receptor binding) was performed. When pretreated with free LA, the fluorescence intensity of the cells with GCGA–siPAK1 (Figure 4A, in the seventh column) decreased substantially, compared to those under normal conditions without excess free LA (Figure 4A, in the fourth column). Moreover, the fluorescence intensity was recovered to approximately the level of the GACS–siPAK1 group (Figure 4A, in the fifth column). These results demonstrate that free LA molecules can prevent the cellular uptake of GCGA–siPAK1 by competitive binding to the ASGP-R receptors on the cell surfaces. We, therefore, directly confirmed that the higher observed efficacy in the cellular uptake of GCGA–siPAK1 compared to GACS–siPAK1 is due to the additional LA ligands on the NP surfaces. Besides, we also determined that the fluorescence intensity of the cells pretreated free GA was obviously decreased compared to those without free GA, suggesting that the targeting capability of GCGA–siPAK1 is higher than that of single-ligand LA-modified NPs (Supplementary Figure 2).

Prior to performing the *in vivo* experiments, the blood compatibility of the NPs, a critical parameter for NP biomedical applications (Yu et al., 2011), was evaluated using hemocompatibility assays. After treatment with various NPs, blood solutions were used to create cell smears to observe the morphological changes in the erythrocyte. Supplementary Figure 3 illustrates that the RBCs remained spherical and intact in both the saline (negative control) and all NP solutions, while a large proportion of RBCs were ruptured in distilled water (positive control). These results suggest that the NPs do not disrupt the RBC membrane integrality and exhibit strong hemocompatibility. To further investigate the impact of the NPs on RBCs, the solutions were centrifugated at the same speed. Figure 4C indicates that no distinct hemolysis was observed in the NP solutions by visual inspection. Moreover, the OD value of the hemoglobin in the supernatant, which implies damaged RBCs, demonstrates that none of the NPs exhibit any remarkable hemolytic activity (Figure 4D). Taken together, all tested NPs possess excellent blood compatibility and could be administered intravenously *in vivo*.

A non-invasive fluorescence imaging system was used to acquire an improved understanding of NP uptake *in vivo*. The tumor-bearing mice were injected with various siPAK1-loaded NPs. The real-time images of the NPs in the entire bodies of live mice were monitored at 8 h after intravenous administration, because this is the optimal time for determining the fluorescence intensity of various organs (Li et al., 2020). It was apparent that all NPs tended to accumulate in the tumor foci rather than normal tissues (Figure 4E). This suggests that the designed NPs offer a mechanism for tumor-specific drug delivery. For mice injected with the dual-targeted GCGA–siPAK1 and GCGA–siNC, the fluorescence signals at the tumor site were the highest compared to other groups. For the mice treated with the GACS–siPAK1, which was decorated with the single-ligand GA, the fluorescence intensity remained elevated in the tumor site. However, the signal was significantly weaker than in those treated with the dual-targeted GCGA–siPAK1. Further, the fluorescence signal of the non-ligand NP, namely CS–siPAK1, was significantly lower than that with the single-ligand. Overall, it is remarkable that the average fluorescence intensities of the tumor tissues obtained from the mice with GCGA–siPAK1 were higher than those treated with GACS–siPAK1, while the fluorescence signals of the mice treated with GACS–siPAK1 were stronger than those treated with CS–siPAK1. These results indicate that GCGA–siPAK1, decorated with two ligands, exhibits the greatest cell targeting capacity, followed by GACS–siPAK1 and GA–siPAK1. Moreover, the mice treated with CS–siPAK1 exhibited weakened fluorescence, even if the NPs were not modified by any ligand, demonstrating that the EPR effect contributed to the selective accumulation of the NPs. Together, the ligand-receptor-mediated endocytosis (active targeting) and EPR effect (passive targeting) both obviously enhance the transportation of siRNA. The GCGA–siPAK1 with a dual-ligand exhibits a superior tumor targeting ability compared to the GACS–siPAK1, which only contains a single moiety. To understand the relative uptake of NPs further, the mice were sacrificed, and various organs and tissues were harvested. As illustrated in Figures 4F,G, the biodistribution of the NPs was consistently low in normal organ tissues, producing a negligible fluorescence signal in all groups, save for the liver and kidneys. As the ASGP-R and GA-R receptors are also expressed in liver cells (He et al., 2010; Pranatharthiharan et al., 2017), the distribution of NPs in the liver appears reasonable. Furthermore, Onishi and Machida reported that water-soluble CS can accumulate in the kidneys for excretion in urine (Onishi and Machida, 1999). This may explain the slight fluorescence intensity in the harvested kidneys.

### GCGA–siPAK1 Suppresses Tumor Growth and Metastasis With Maximum Efficiency

**Figure 5:**
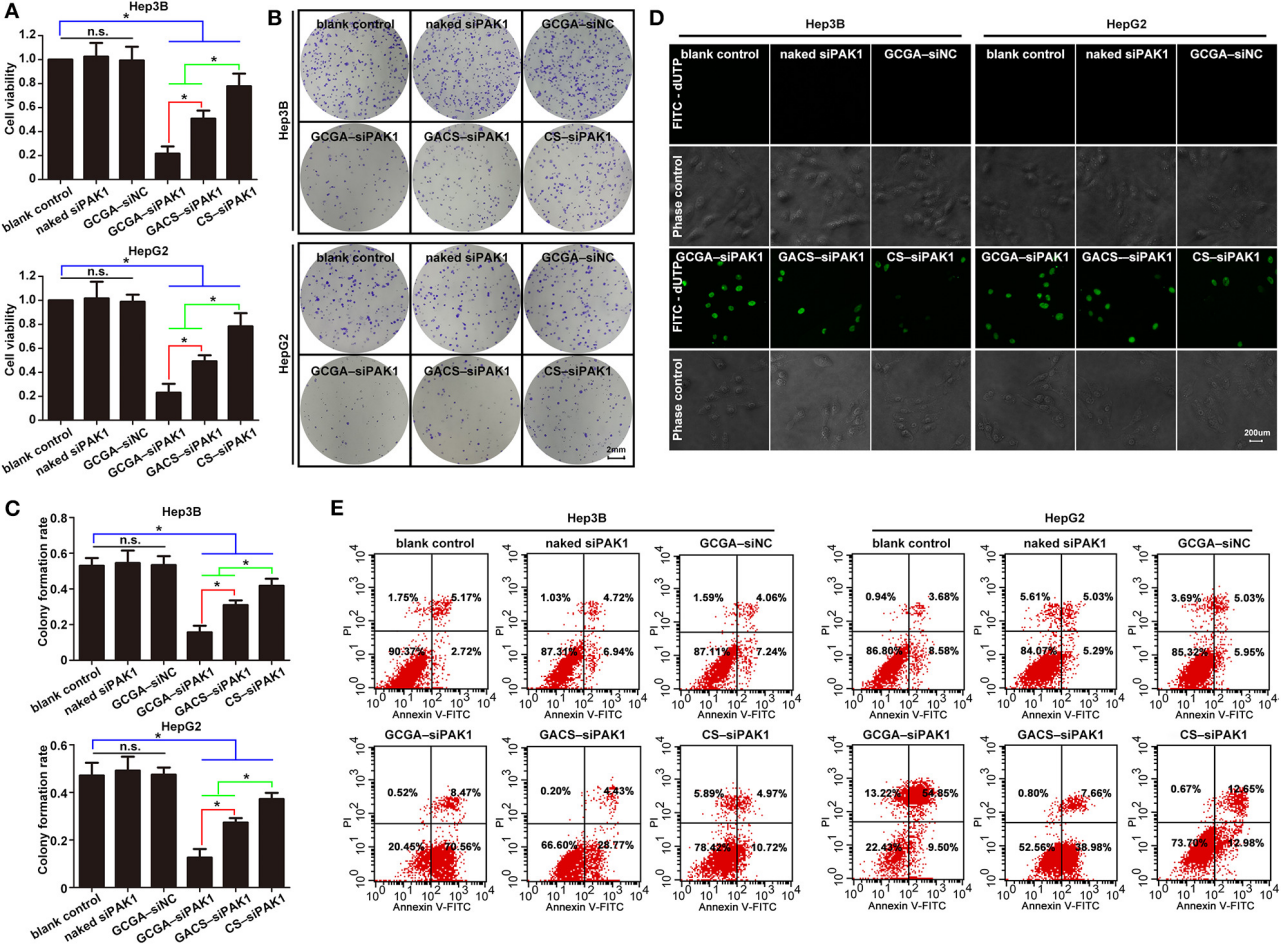
GCGA–siPAK1 suppressed cell proliferation and promoted cell apoptosis with maximum efficiency. **(A)** The cell viability was evaluated using CCK8 assaying after treatment. Data represents the mean ± SD; ^*^P < 0.05; N.S., not significant. **(B)** The colony-forming ability was measured by colony-forming assaying after treatment. **(C)** Statistical analysis of colony-forming efficiency according to **(B)**. Data represents the mean ± SD; ^*^P < 0.05; N.S., not significant. **(D)** Cell apoptosis as examined by the One Step TUNEL Apoptosis Assay Kit. **(E)** Cell apoptosis as determined by flow cytometry assays using the Annexin V-FITC Apoptosis Detection Kit.

After investigating the targeting ability of the NPs, we next determined their anti-tumor effects. Although the dual-targeted GCGA–siNC, carrying a nonsense nucleotide sequence, exhibited the greatest targeting ability and siRNA transportation efficacy, it did not exhibit a significant decrease in cell viability or colony-forming efficiency (Figures 5A–C). Moreover, only a slight decrease in cell proliferation was observed in the CS–siPAK1 group, while a greater decrease was exhibited in the GACS–siPAK1 group compared to the blank control group, indicating that the GA-decorated NPs exhibit greater repression of cell proliferation than the non-ligand NP. More importantly, the GCGA–siPAK1 with dual-ligands exhibited the greatest decrease in cell proliferation compared to the others, indicating the dominance of GCGA–siPAK1 in inhibiting cell proliferation.

Then, the effects of the NPs on cell apoptosis were investigated. As illustrated in Figures 5D,E, the percentage of apoptotic cells was markedly increased after treatment with GCGA–siPAK1, GACS–siPAK1, and CS–siPAK1 compared to the blank control, while no difference was observed between the blank control, naked siPAK1, and GCGA–siNC groups. It is, thus, apparent that all siPAK1-loaded NPs exhibit the capability of promoting HCC cell apoptosis *in vitro*. Furthermore, for the cells treated with GACS–siPAK1, the percentage of apoptotic cells was significantly higher than for those treated with CS–siPAK1, yet significantly lower than the others treated with GCGA–siPAK1. Hence, GCGA–siPAK1 exhibits the strongest performance in promoting cell apoptosis.

**Figure 6:**
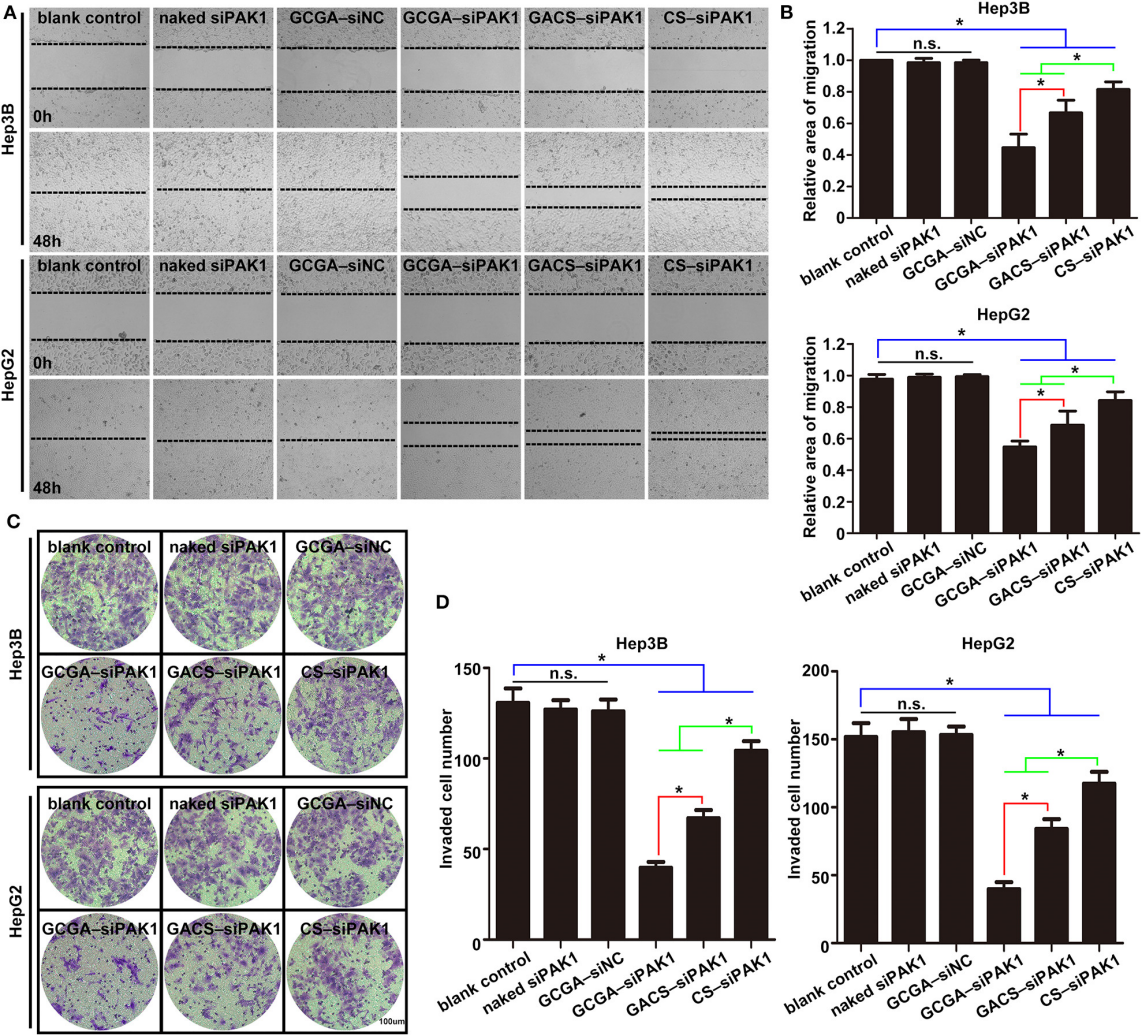
GCGA–siPAK1 suppression of cell migration and invasion with greatest efficiency. **(A)** Cell migration ability was evaluated by a wound-healing assay after treatment. **(B)** Statistical analysis of the cell migration rate according to **(A)**. Data represents the mean ± SD; ^*^P < 0.05; N.S., not significant. **(C)** The cell invasiveness was evaluated by Transwell assay after treatment. **(D)** Statistical analysis of cell invasion rate according to **(C)**. Data represents the mean ± SD; ^*^P < 0.05; N.S., not significant.

Cell migration and invasion were examined using wound-healing assays and Transwell invasion assays individually. As illustrated in Figure 6, the healing rates and number of invaded cells in the blank control group were significantly higher than those of the experimental groups (including GCGA–siPAK1, GACS–siPAK1, and CS–siPAK1), indicating that all siPAK1-loaded NPs could suppress HCC cell migration and invasion *in vitro*. For the cells treated with GACS–siPAK1, the healing rates and number of invaded cells were both lower than those treated with CS–siPAK1, however, both were significantly higher than in others treated with GCGA–siPAK1. These results indicate that the GCGA–siPAK1 exhibits the greatest inhibitory efficacy on tumor cell metastasis.

Overall, the GCGA–siNC did not affect the HCC cells in terms of any biological behaviors, suggesting that the dual-ligand modified NPs exhibited strong biocompatibility *in vitro*, and the siRNA of nonsense sequences would not disturb the cellular environmental homeostasis. The GCGA–siPAK1 and GACS–siPAK1 were both superior to the CS–siPAK1 in inhibiting the tumor cell growth and metastasis. This is consistent with the results of previous studies (Tian et al., 2010; Craparo et al., 2014), suggesting that the specific ligand-modified NPs enhance the association of hepatoma cells by means of the receptor-mediated mechanism, followed by a stronger tumor repression effect. More notably, the GCGA–siPAK1 was greater than the GACS–siPAK1, indicating that the dual-modified NPs were superior to the single-modified NPs in their affinity of liver cancer and tumor inhibition ability.

### GCGA–siPAK1 Inhibits Expression of PAK1 in HCC Cells

**Figure 7:**
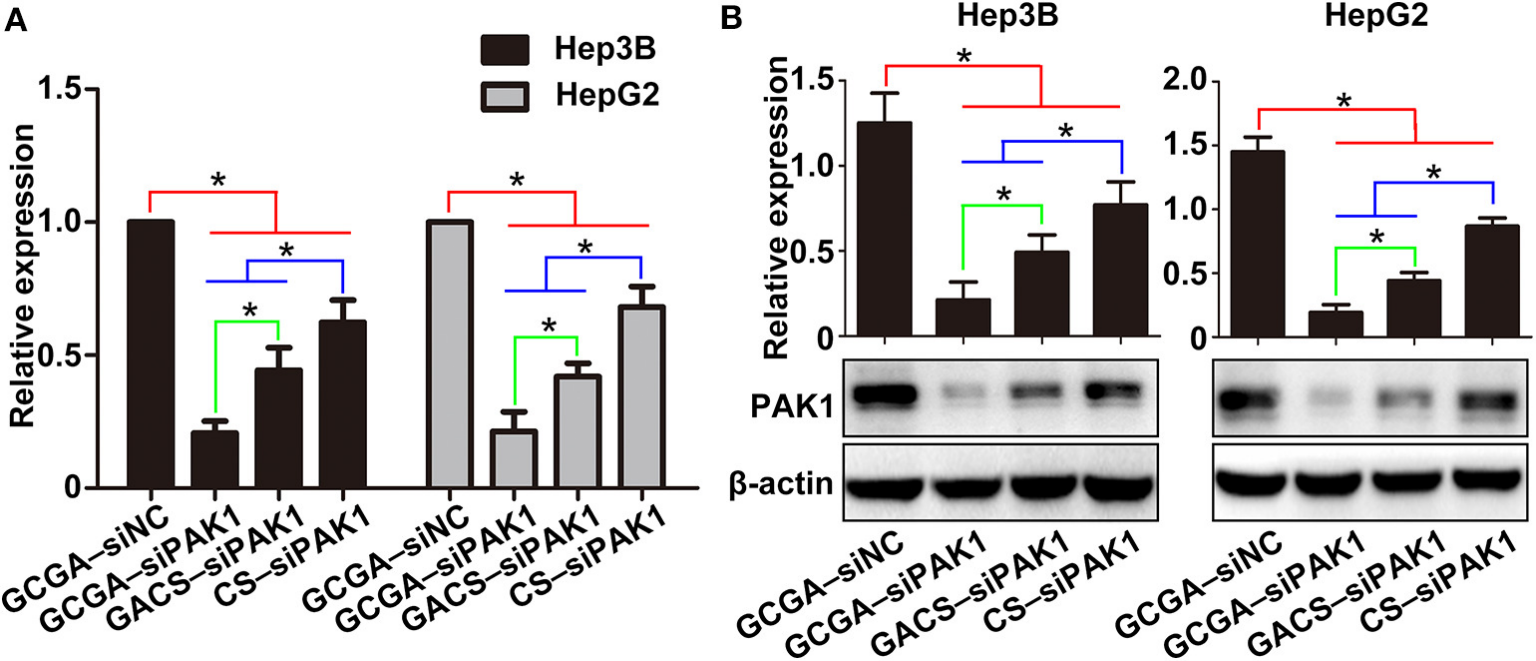
GCGA–siPAK1 inhibition of PAK1 expression in HCC cells. **(A)** PAK1 mRNA expression in HCC cell lines after treatment. **(B)** Protein expression of PAK1 measured by western blot analysis. Data represents the mean ± SD; ^*^P < 0.05.

To explore the mechanism responsible for the GCGA–siPAK1-induced tumor repression, we quantified the expression of PAK1 at the mRNA and protein levels. Considering that the GCGA–siNC, as with the blank control and naked siPAK1, did not exhibit any influence on the cellular biological behaviors, we designated it as the control group in the following experiments. As illustrated in Figure 7A, the cells treated with siPAK1-loaded NPs all exhibited a PAK1-silencing effect with various intensities. These results suggest that the siPAK1, and not the polymer backbone of the CS or ligands, is the key feature required for the inhibition of liver cancer *in vitro*. Moreover, the cells treated with GCGA–siPAK1 exhibited the lowest mRNA expression of PAK1 in all groups, indicating that the GCGA–siPAK1 demonstrated optimal PAK1-silencing effects. The western blot analysis provided similar results, with the PAK1 protein expression lowest in cells with GCGA–siPAK1, followed by GACS–siPAK1 and CS–siPAK1 (Figure 7B). Combined with the previous results (Figures 4–6), we demonstrate that the quantity of the siPAK1 transported into the cells dominates the PAK1-silencing efficiency and plays a pivotal role in tumor repression.

### Molecular Mechanism Responsible for Pro-apoptotic Properties of GCGA–siPAK1 in HCC Cells

**Figure 8:**
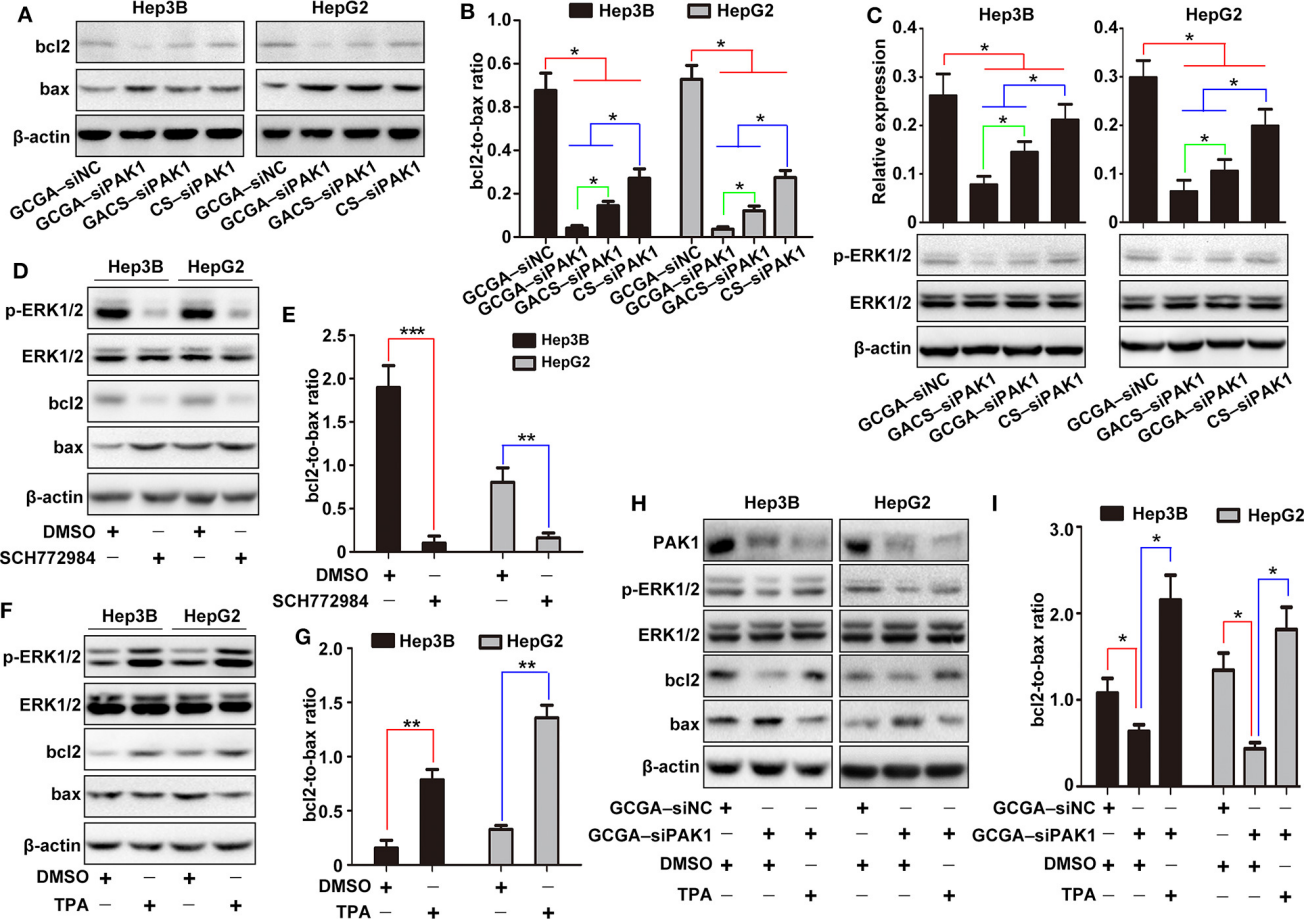
Molecular mechanism of GCGA–siPAK1-induced cell apoptosis in HCC cells. **(A)** Expression of bcl2 and bax measured by western blot. **(B)** Ratio of bcl2 to bax calculated according to **(A)**. Data represents the mean ± SD; ^*^P < 0.05. **(C)** Expression of p-ERK1/2. The ERK1/2 protein was measured as an internal reference. Data represents the mean ± SD; ^*^P < 0.05. **(D)** Expression of p-ERK1/2, bcl2, and bax following treatment with the p-ERK1/2 inhibitor (SCH772984) for 24 h. The ERK1/2 protein was measured as an internal reference. **(E)** Expression ratio of bcl2 to bax calculated according to **(D)**. Data represents the mean ± SD; ^**^P < 0.01 and ^***^P < 0.001. **(F)** Expression of p-ERK1/2, bcl2, and bax following treatment with p-ERK1/2 activator (TPA) for 24 h. The ERK1/2 protein was measured as an internal reference. **(G)** Expression ratio of bcl2 to bax calculated according to **(F)**. Data represents the mean ± SD; ^**^P < 0.01. **(H)** Cells pretreated with or without TPA were incubated in the NPs. The protein expressions of PAK1, p-ERK1/2, bcl2, and bax were then measured. **(I)** Expression ratio of bcl2 to bax calculated according to **(H)**. Data represents the mean ± SD; ^*^P < 0.05.

To investigate the molecular mechanism whereby GCGA–siPAK1 promotes cell apoptosis, we introduced bcl2 family members, which are widely associated with the regulators of cell death through the endogenous apoptotic pathways. Bcl2 is one of most significant regulatory antiapoptotic factors, while bax is an important proapoptotic factor in the bcl2 family. Moreover, the bcl2:bax expression ratio is critical for determining endogenous cell apoptosis (Korsmeyer et al., 1993). As illustrated in Figures 8A,B, the protein expressions of bcl2 and bax were determined, and the bcl2:bax ratios were calculated. Compared to GCGA–siNC, the bcl2:bax ratio in the GCGA–siPAK1, GACS–siPAK1, and CS–siPAK1 groups all decreased significantly, indicating that the siPAK1-loaded NPs promote cell apoptosis. Moreover, the cells treated with GCGA–siPAK1 exhibited the lowest bcl2:bax ratio. That is, the GCGA–siPAK1 apparently promotes cell apoptosis with the highest efficiency, which is consistent with our previous findings (Figures 5D,E).

Extracellular signal-regulated kinases (ERK1/2s) are widely expressed in differentiated cells and involved in various cellular functions. Numerous studies have revealed that p-ERK1/2 (T202/Y204), as one of the most important molecules in the MEK/ERK pathway, can be regulated by PAK1 to influence cell migration and proliferation in various cancers (Du et al., 2010; El-Baba et al., 2014). However, whether PAK1 regulates cell apoptosis *via* p-ERK1/2 is unclear. Hence, we determined the protein expression of p-ERK1/2 for investigating its role in cell apoptosis. As illustrated in Figure 8C, the expression of p-ERK1/2 was lowest in cells with GCGA–siPAK1, followed by GACS–siPAK1 and CS–siPAK1. Thus, we can readily observe that p-ERK1/2 expression exhibited the same tendency as the PAK1 protein expression in those groups (Figure 7B), demonstrating a strong correlation with the GCGA–siPAK1-induced biological behaviors.

To further investigate whether the MEK/ERK pathway plays an important role in the GCGA–siPAK1-induced cell apoptosis, the inhibitor and activator of p-ERK1/2 were used. After cells were treated with the p-ERK1/2 inhibitor, SCH772984, the expressions of p-ERK1/2 and bcl2 decreased, while that of bax increased (Figure 8D). Moreover, the bcl2:bax expression ratio significantly decreased (Figure 8E). These results reveal that cell apoptosis would be promoted by decreasing the expression of p-ERK1/2. Alternatively, the expression of p-ERK1/2 and bcl2 increased while that of bax decreased after cells were treated with the p-ERK1/2 activator, TPA (Figure 8F). Furthermore, the bcl2:bax expression ratio was significantly increased (Figure 8G). These results indicate that HCC cell apoptosis is effectively repressed by elevating the expression of p-ERK1/2; while also verify the validity of TPA for the following experiments. Furthermore, following pretreatment with TPA for 24 h, the cells treated with GCGA–siPAK1 exhibited an increase in p-ERK1/2 expression (Figure 8H). Similarly, the bcl2:bax expression ratio was distinctly increased (Figure 8I). These results demonstrate that the p-ERK1/2 activator reverses the bcl2:bax expression ratio and inhibits GCGA–siPAK1-induced cell apoptosis. Overall, GCGA–siPAK1 promotes HCC cell apoptosis by decreasing the expression of p-ERK1/2. We, therefore, directly described the cell apoptosis mechanism whereby GCGA–siPAK1 triggers the endogenous apoptotic pathways, which is regulated by bcl2 and bax, through the PAK1/MEK/ERK pathway.

### GCGA–siPAK1 Inhibits HCC Cells *in vivo*

**Figure 9:**
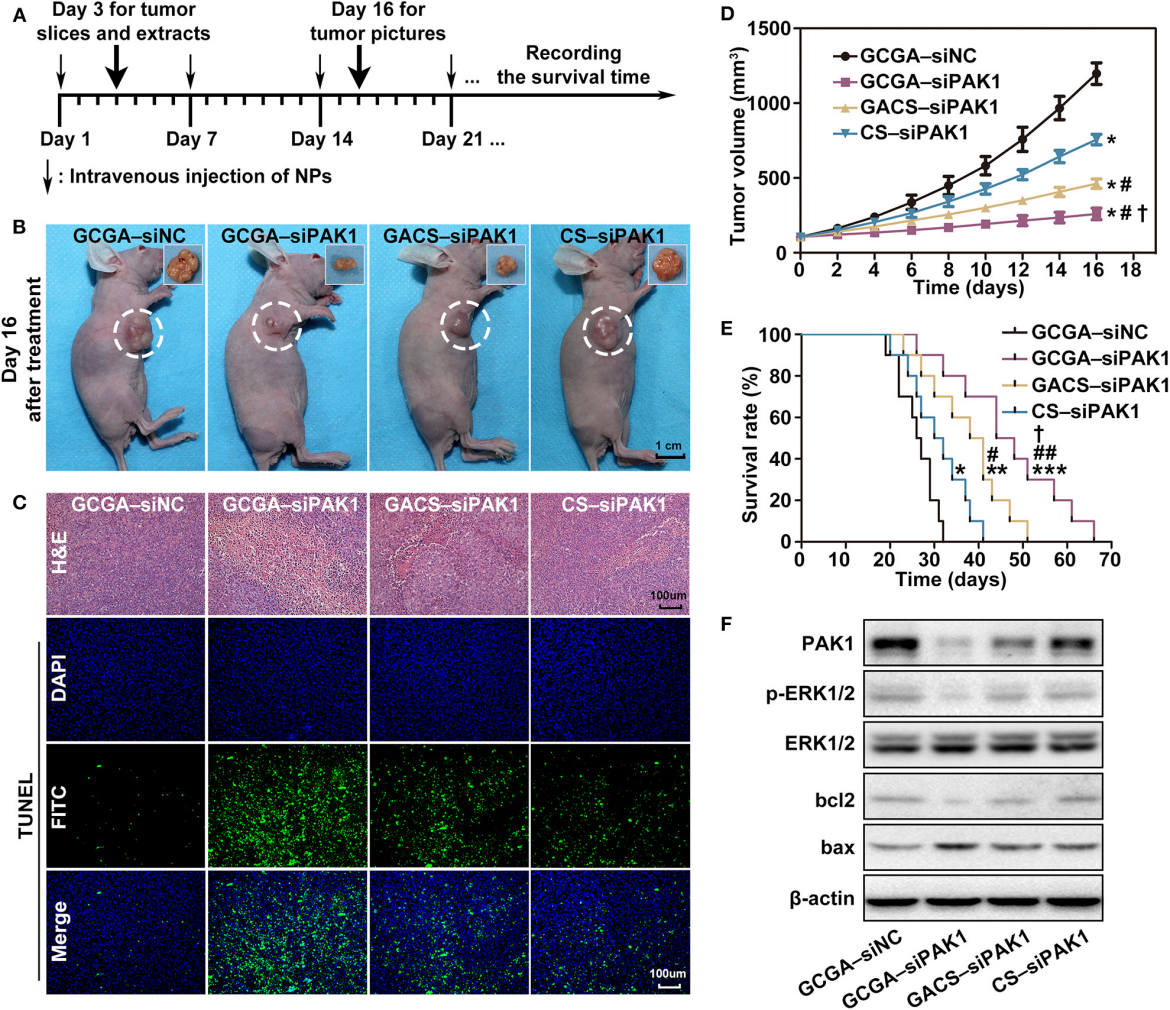
Inhibition of GCGA–siPAK1 for HCC *in vivo*. **(A)** Timeline for the assessment of the antitumor activities of the NPs in subcutaneous xenograft model. **(B)** Antitumor effect *in vivo*; photographs of xenografted tumors on day 16 after treatment. **(C)** H&E staining for pathological changes in tumor sections (top row). TUNEL staining (green) for apoptosis in tumor sections (three bottom rows). Blue fluorescence localized in the cell nuclei. **(D)** Tumor volume growth curves at different time points following treatments in four groups (*n* = 4 per group). Data represents the mean ± SD; ^*^P < 0.05, compared with GCGA–siNC group; ^#^*P* < 0.05, compared with CS-siPAK1 group; and ^†^*P* < 0.05, compared with GACS–siPAK1 group. **(E)** Survival curves of mice in four groups with different treatments (*n* = 10 per group). ^*^P < 0.05, ^**^P < 0.01, and ^***^P < 0.001, compared with GCGA–siNC group; ^#^*P* < 0.05 and ^##^*P* < 0.01, compared with CS–siPAK1 group; and ^†^*P* < 0.05, compared with GACS–siPAK1 group. **(F)** Protein expressions of PAK1, p-ERK1/2, bcl2, and bax in tumor tissues.

To further examine the therapeutic efficacy of the siPAK1-loaded NPs against HCC *in vivo*, tumor mouse models were injected intravenously with 100 μL NPs solution once a week (Figure 9A). As illustrated in Figure 9B, significant differences in the tumor shrinkage were observed among all experimental groups (treatment with GCGA–siPAK1, GACS–siPAK1, and CS–siPAK1) and the control group (treatment with GCGA–siNC) on day 16 after intravenous administration. The tumor shrinkage in the GCGA–siPAK1 group was significantly greater than that of the other groups. These results were further verified by quantitative determination of the tumor volume. Figure 9D illustrates that the siPAK1-loaded NPs inhibited the tumor growth, particularly in the GCGA–siPAK1, with the highest efficiency. Furthermore, the histomorphology and percentage of apoptotic cells in the tumor tissue were detected using H&E staining and TUNEL assay, revealing that GCGA–siPAK1 induced the highest levels of cell death and apoptosis (Figure 9C). More importantly, the dual-ligand modified GCGA–siPAK1 apparently prolonged the survival time of the mice and improved the long-term outcome for HCC *in vivo* compared to the other groups (Figure 9E). In particular, the tumor-bearing control mice began to die on the 19th day after treatment, and all mice had died by the 32nd day, with a median survival time of 26 days. However, the tumor-bearing mice in the GCGA–siPAK1 group began to die on the 26th day and all mice had died by the 66th day, with a median survival time of 44 days, which was also longer than those in the GACS–siPAK1 group (median survival time of 38 days) and CS–siPAK1 group (median survival time of 30 days). To investigate the molecular regulation mechanism *in vivo*, the proteins of the xenografted tumor were extracted. The expression of PAK1, p-ERK1/2, and bcl2 in cells treated with the siPAK1-loaded NPs were all decreased, while the expression of bax protein was increased (Figure 9F and Supplementary Figure 5). These results suggest that GCGA–siPAK1 promotes tumor apoptosis through down-regulation of the bcl2:bax expression ratio *via* the PAK1/MEK/ERK pathway *in vivo*, which is consistent with *in vitro* experiments.

### Systemic Toxicity Evaluation *in vivo*

**Figure 10:**
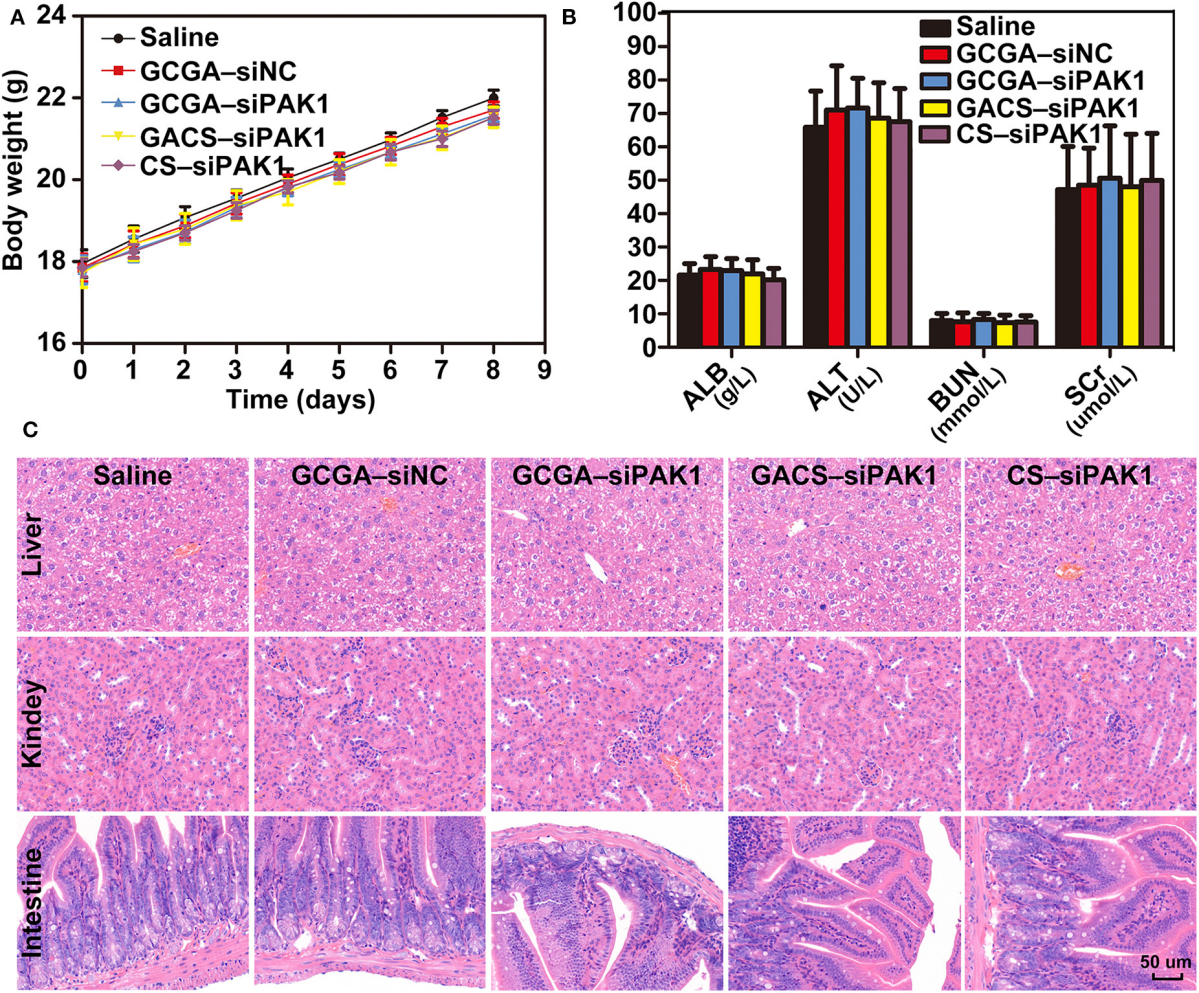
Systemic toxicity evaluation *in vivo*. **(A)** Body weight changes in BALB/c mice after respective treatments. Data represents the mean ± SD. **(B)** Blood biochemistry analysis of mice injected with NPs. Data represents the mean ± SD. **(C)** Histopathologic images (H&E staining, 400×) of various organ sections in mice on the eighth day following treatments.

To exploit the potential systemic toxicity of the NPs, a double dose of treatment was injected into healthy mice *via* the tail vein. As illustrated in Figure 10A, regardless of the treatments, no significant changes in body weight were observed and all rats demonstrated weight gains at a similar rate. During the entire experimental period, no mortality, or abnormal performance was observed in any of the groups, and all rats maintained normal activity levels and a healthy appearance. Subsequently, the biochemical parameters, such as hepatic function biomarkers, namely albumin (ALB) and alanine transaminase (ALT), and renal-related indicators, namely blood urea nitrogen (BUN) and serum creatinine (Cr), were evaluated. As illustrated in Figure 10B, these indicators were all at normal levels with no significant differences observed in any group, indicating strong safety profiles for these NPs within the liver and kidney. Moreover, we conducted a histological analysis for evaluating the potential tissue toxicity of the NPs. According to the H&E staining examination, no unusual pathological changes, particularly inflammatory cell infiltrates or tissue damage, were observed in any of the organs following NP administrations (Figure 10C and Supplementary Figure 4). Overall, the NPs exhibit excellent biocompatibility in mice, which provides adequate evidences for clinical applications.

## Conclusion

In summary, we developed a novel dual-targeted drug delivery system, namely the GCGA–siPAK1. Owing to the active targeting capacity of GA and LA, this delivery system remarkably enhances the cellular uptake and promotes gene delivery to HCC cells *in vitro* and *in vivo*. Furthermore, we found that it exhibits strong antitumor effects without eliciting systemic toxic side effects. Finally, we directly elucidated the molecular mechanism employed by GCGA–siPAK1 in promoting cell apoptosis. Overall, this novel dual-modified NP can effectively promote the therapeutic effect of HCC and provides a promising gene therapy strategy for future clinical oncotherapy.

## Data Availability Statement

All datasets generated for this study are included in the article/Supplementary Material.

## Ethics Statement

The animal study was reviewed and approved by Animal Care Committee at Tongji Medical College.

## Author Contributions

SJ, ML, and QZ: conceptualization. SJ, YW, DS, YZ, and SH: data curation. SJ, CZ, PS, XC, SH, and ZS: formal analysis. ML, ZS, and QZ: funding acquisition. SJ and SH: investigation. SJ, YG, and XC: methodology. SJ and QZ: project administration. ML, ZS, and QZ: resources. YW, CZ, YZ, ML, and XC: software. ML, SH, ZS, and QZ: supervision. XC, SH, ZS, and QZ: validation. SJ: visualization. SJ and ML: writing original draft. All authors: listed have approved this manuscript.

## Conflict of Interest

The authors declare that the research was conducted in the absence of any commercial or financial relationships that could be construed as a potential conflict of interest.
