# Corrigendum: Dual-Targeting Nanoparticle-Mediated Gene Therapy Strategy for Hepatocellular Carcinoma by Delivering Small Interfering RNA

**DOI:** 10.3389/fbioe.2021.656268

**Published:** 2021-02-11

**Authors:** Qi Chang Zheng, Shuai Jiang, Yu Zhe Wu, Dan Shang, Yong Zhang, Shao Bo Hu, Xiang Cheng, Chen Zhang, Ping Sun, Yang Gao, Zi Fang Song, Min Li

**Affiliations:** ^1^Department of Hepatobiliary Surgery, Union Hospital, Tongji Medical College, Huazhong University of Science and Technology, Wuhan, China; ^2^Department of Vascular Surgery, Union Hospital, Tongji Medical College, Huazhong University of Science and Technology, Wuhan, China

**Keywords:** small interfering RNA, gene therapy, targeted therapy, chitosan, hepatocellular carcinoma, drug delivery

In the published article, there was an error in affiliations 1 and 2. Affiliation 1 should be “Department of Hepatobiliary Surgery, Union Hospital, Tongji Medical College, Huazhong University of Science and Technology, Wuhan, China”. Affiliation 2 should be “Department of Vascular Surgery, Union Hospital, Tongji Medical College, Huazhong University of Science and Technology,Wuhan, China”.

In the original article, there was a mistake in the Graphical Abstract and in Figure 1 as published. “EPR effect” was incorrectly written as “ERP effect”. The corrected Graphical Abstract and Figure 1 appear below.

**Graphical Abstract:**
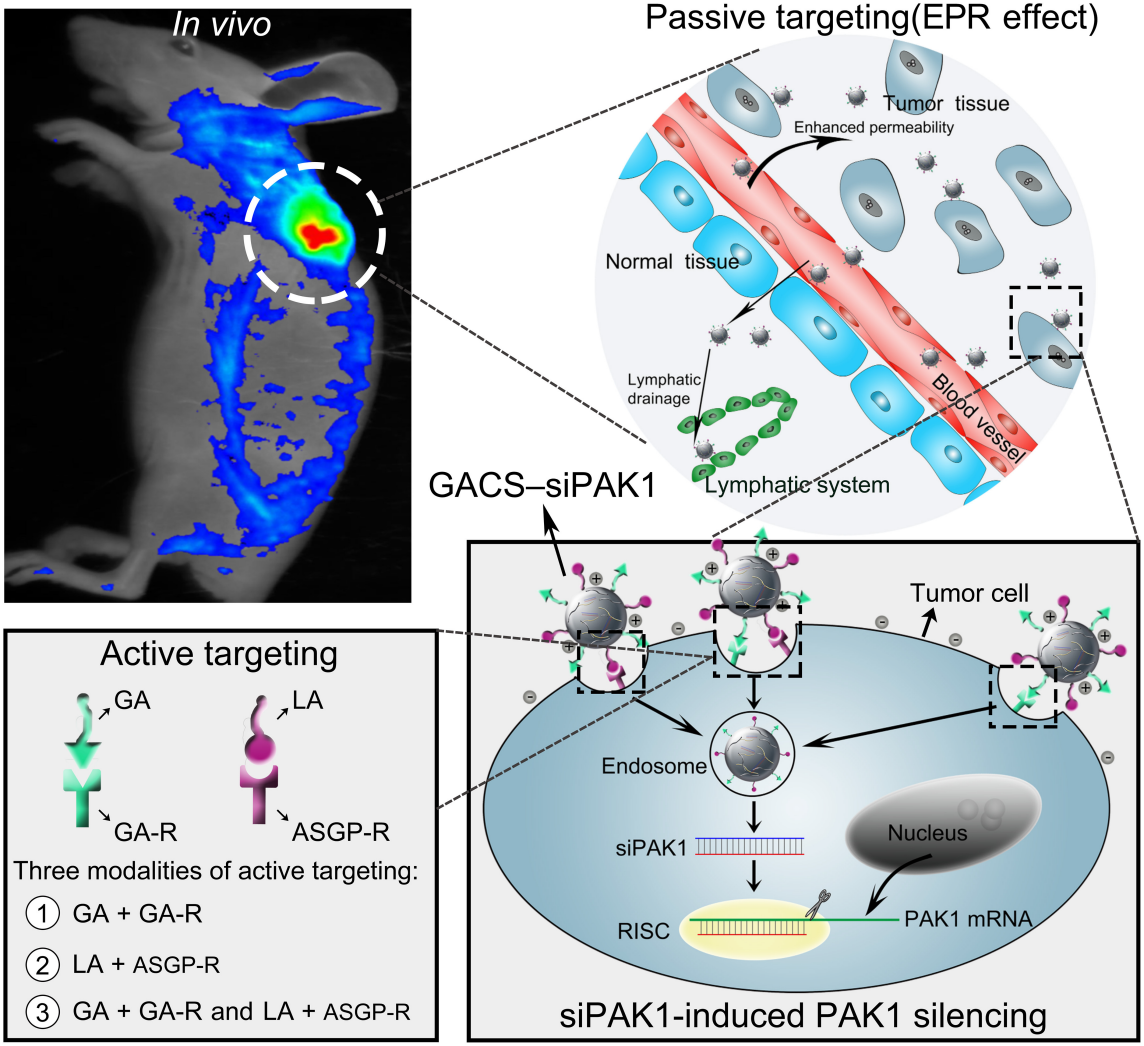


**Figure 1:**
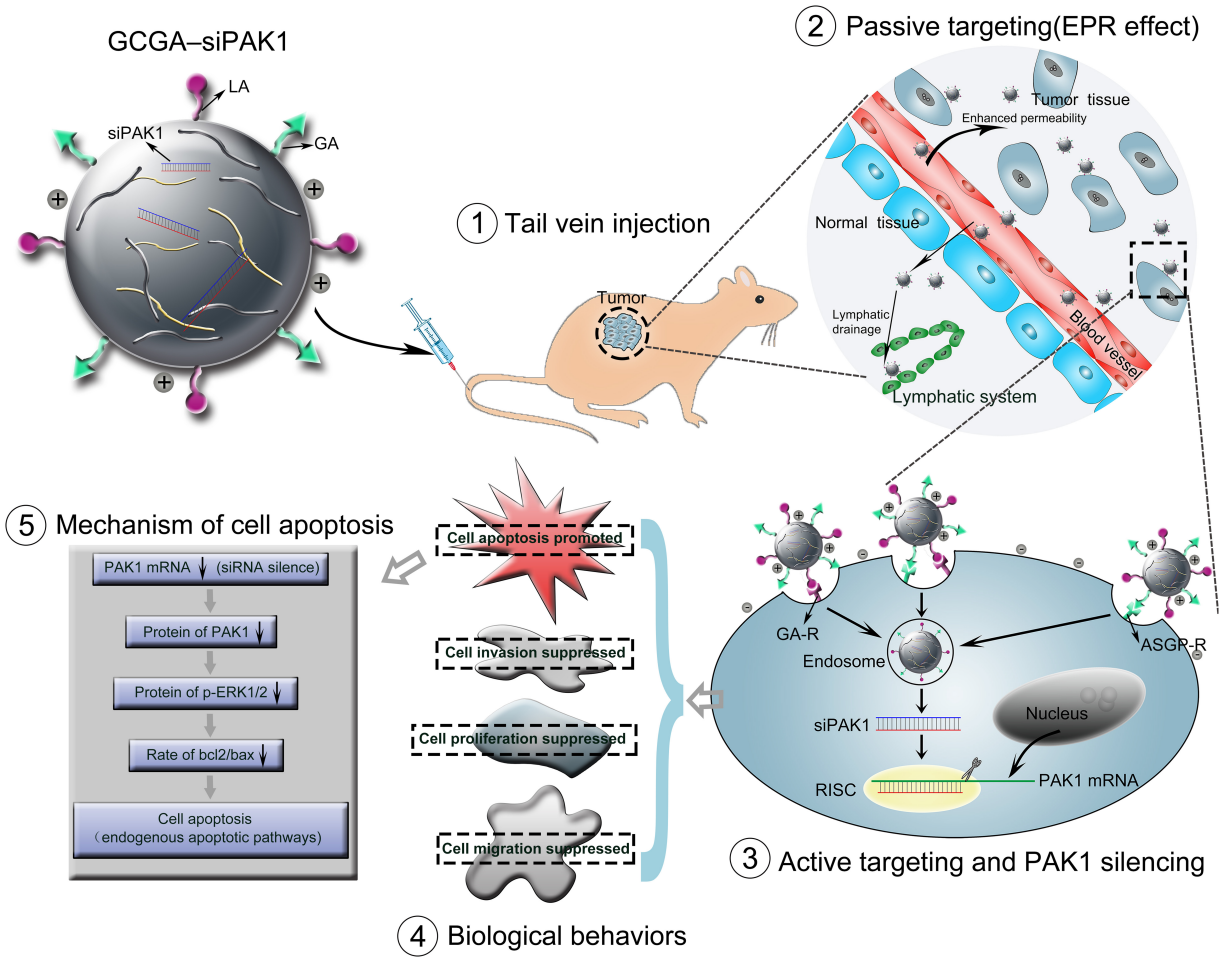
Schematic representation of GCGA–siPAK1 promoting targeted delivery and therapeutic efficacy in HCC xenograft mouse model. The process includes four steps: (1) intravenous administration of GCGA–siPAK1 *via* tail vein; (2) NPs accumulation in tumor tissue *via* passive targeting (commonly known as the EPR effect); (3) three modalities of active targeting *via* dual-ligand-receptor-mediated endocytosis and mechanism of RNAi (siPAK1-induced PAK1 silencing); (4) tumor biological behaviors after PAK1 silencing; and (5) molecular mechanism of promoting cell apoptosis *via* PAK1/MEK/ERK pathway.

The authors apologize for this error and state that this does not change the scientific conclusions of the article in any way. The original article has been updated.

