# Supplementary material for: Dual-Targeting Nanoparticle-Mediated Gene Therapy Strategy for Hepatocellular Carcinoma by Delivering Small Interfering RNA
Source: Front Bioeng Biotechnol. 2020 Jun 10;8:512. doi: 10.3389/fbioe.2020.00512 (PMC7297947; doi:10.3389/fbioe.2020.00512)
Supplement: Supplementary file 1 [file Data_Sheet_1.pdf]

Supplementary Material

Dual-targeting Nanoparticle-mediated Gene Therapy Strategy for Hepatocellular Carcinoma by Delivering Small Interfering RNA

Qi Chang Zheng[1†], Shuai Jiang[1†], Yu Zhe Wu[1], Dan Shang[2], Yong Zhang[1], Shao Bo Hu[1], Xiang Cheng[1], Chen Zhang[1], Ping Sun[1], Yang Gao[1], Zi Fang Song[1*], Min Li[1*]

[1]Department of Hepatobiliary Surgery, Union Hospital, Tongji Medical College, Huazhong University of Science and Technology, Wuhan, China

[2]Department of Vascular Surgery, Union Hospital, Tongji Medical College, Huazhong University of Science and Technology, Wuhan, China

*** Correspondence:**
Min Li, liminmed@hust.edu.cn; Zi Fang Song, zsong@hust.edu.cn

† These authors contributed equally to this work.

Figures

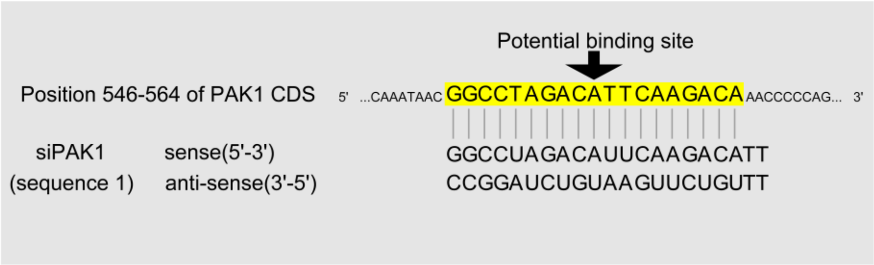

Supplementary Figure S1. Potential siPAK1 binding site in PAK1 sequence.

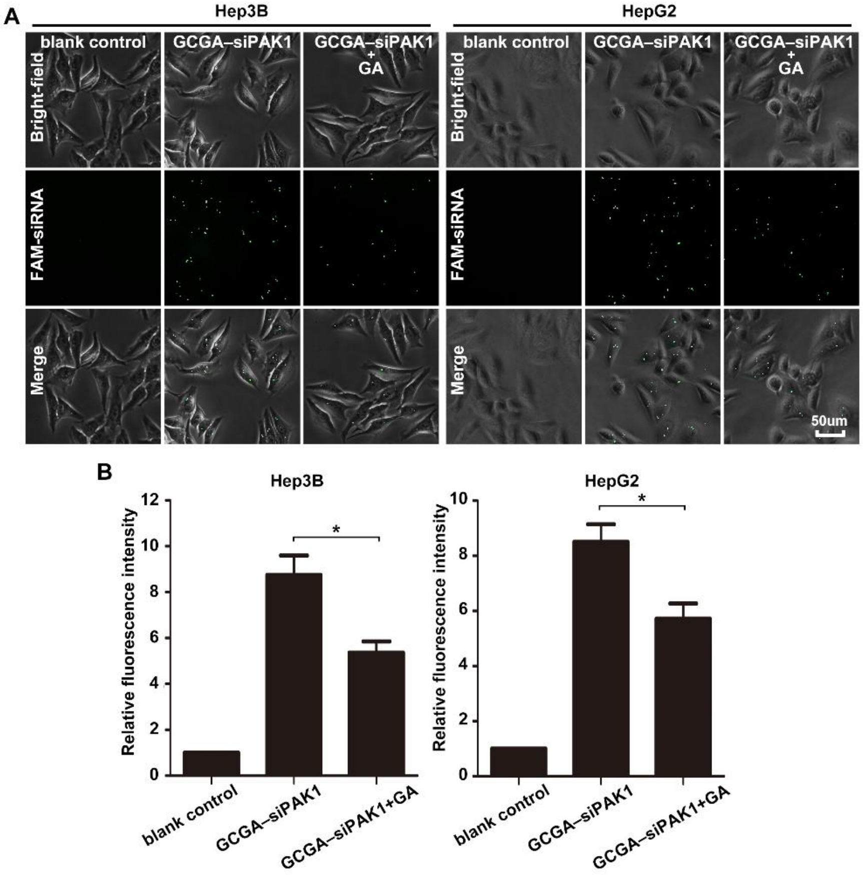

Supplementary Figure S2. (A) Fluorescence images of GCGA–siPAK1 in HCC cells pretreated with free GA (100 ug/mL)

for 30 min. (B) Statistical analysis of fluorescence intensity according to (A).

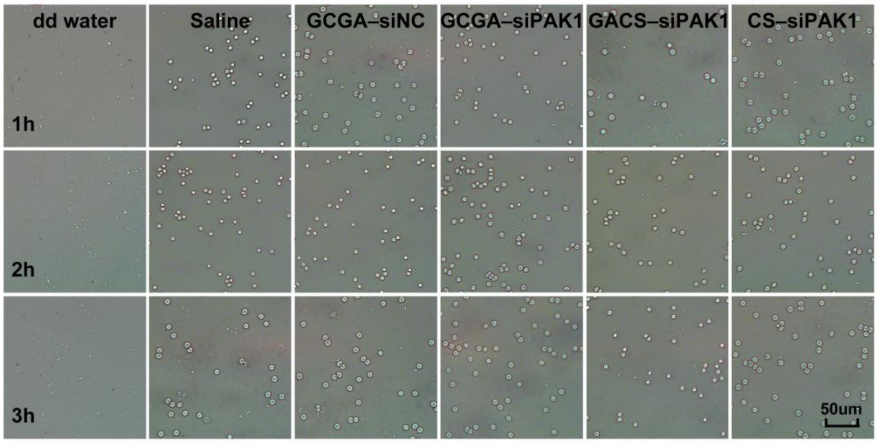

Supplementary Figure S3. Hemolysis assay of RBCs. RBCs were incubated with different NP formulations for 1, 2 and 3 h, respectively. Optical microscopic observations of erythrocytes after incubation with distilled water, saline, and NPs.

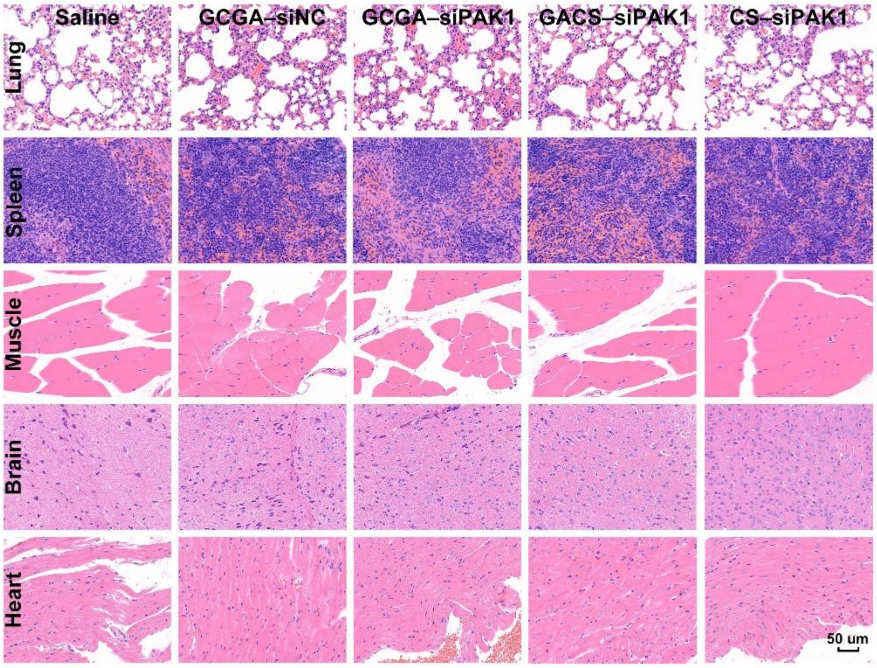

Supplementary Figure S4. Histopathologic images (400×) with H&E staining. Various organ sliced from mice on the

eighth day following different treatments.

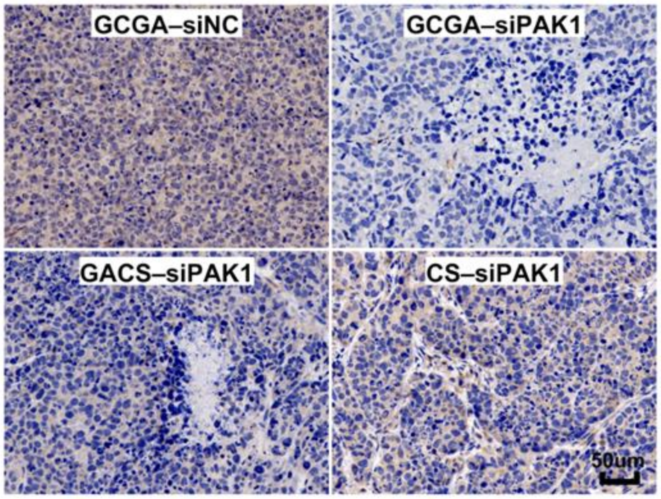

Supplementary Figure S5. The immunohistochemical staining of PAK1 in tumor sections.
